# γ-Secretase modulator resistance of an aggressive Alzheimer-causing presenilin mutant can be overcome in the heterozygous patient state by a set of advanced compounds

**DOI:** 10.1186/s13195-025-01680-3

**Published:** 2025-02-19

**Authors:** Johannes Trambauer, Rosa Maria Rodriguez Sarmiento, Holly J. Garringer, Katja Salbaum, Liliana D. Pedro, Dennis Crusius, Ruben Vidal, Bernardino Ghetti, Dominik Paquet, Karlheinz Baumann, Lothar Lindemann, Harald Steiner

**Affiliations:** 1 Division of Metabolic Biochemistry, Faculty of Medicine, Biomedical Center (BMC), LMU Munich, Feodor-Lynen-Str. 17, Munich, 81377 Germany; 2 German Center for Neurodegenerative Diseases (DZNE), Munich, 81377 Germany; 3 Pharma Research and Early Development, F. Hoffmann-La Roche AG, Therapeutic Modalities, Small Molecule Research, Roche Innovation Center Basel, Basel, 4070 Switzerland; 4 Department of Pathology and Laboratory Medicine, Indiana University School of Medicine, Indianapolis, IN 46202 USA; 5 Institute for Stroke and Dementia Research, University Hospital, LMU Munich, Munich, 81377 Germany; 6 Munich Cluster of Systems Neurology (SyNergy), Munich, 81377 Germany; 7 Pharma Research and Early Development, F. Hoffmann-La Roche AG, Neuroscience and Rare Diseases Translational Area, Neuroscience Discovery, Roche Innovation Center Basel, Basel, 4070 Switzerland

**Keywords:** Alzheimer’s disease, γ-Secretase modulator, Aβ, Presenilin, Familial Alzheimer’s disease

## Abstract

**Background:**

Amyloid-β peptide (Aβ) species of 42 or 43 amino acids in length (Aβ42/43) trigger Alzheimer´s disease (AD) and are produced in abnormal amounts by mutants of the γ-secretase subunit presenilin-1 (PS1), which represent the primary cause of familial AD (FAD). Lowering these peptides by γ-secretase modulators (GSMs) is increasingly considered a safe strategy to treat AD since these compounds do not affect the overall cleavage of γ-secretase substrates. GSMs were shown to modulate not only wild-type (WT) γ-secretase but also FAD mutants, expanding their potential use also to the familial form of the disease. Unlike most other FAD mutants, the very aggressive PS1 L166P mutant is largely resistant to GSMs. However, these data were mostly obtained from overexpression models, which mimic more the less relevant homozygous state rather than the heterozygous patient situation.

**Methods:**

Mouse embryonic fibroblast and induced pluripotent stem cell-derived neuronal PS1 L166P knock-in (KI) cell models were treated with various GSMs and Aβ responses were assessed by immunoassays and/or gel-based analysis.

**Results:**

We identified GSMs that lower Aβ42 and/or Aβ43 when PS1 L166P is heterozygous, as it is the case in affected patients, and could reduce the amount of pathogenic Aβ species towards WT levels. RO7019009 was the most potent of these compounds, reducing both pathogenic species and concomitantly increasing the short Aβ37 and Aβ38, of which the latter has been associated with delayed AD progression. Another effective compound, the structurally novel indole-type GSM RO5254601 specifically acts on the Aβ42 product line leading to a selective increase of the beneficial Aβ38. Interestingly, we further found that this class of GSMs can bind not only one, but both presenilin fragments suggesting that it targets γ-secretase at an unusual binding site.

**Conclusion:**

Our data show that even highly refractory presenilin FAD mutants are in principle tractable with GSMs extending the possibilities for potential clinical studies in FAD with suitable GSM molecules.

**Supplementary Information:**

The online version contains supplementary material available at 10.1186/s13195-025-01680-3.

## Background

Accumulation and deposition of amyloid-β peptide (Aβ) species in the brain is a major hallmark of Alzheimer´s disease (AD) [1]. The longer Aβ42 and Aβ43 species are believed to trigger a cascade of pathogenic events that ultimately lead to neurodegeneration and dementia in AD patients [2]. Aβ is generated by secretases, proteases that sequentially cleave the β-amyloid precursor protein (APP) [3]. After removal of most of the APP ectodomain by β-secretase, the transmembrane domain of the resultant APP C-terminal fragment (CTF) is proteolytically attacked by γ-secretase complexes. After initial cleavage by the catalytic subunit presenilin-1 (PS1) or PS2 close to the cytosolic membrane border, subsequent stepwise carboxy-terminal trimming releases Aβ species of varying lengths [4]. This so-called processivity of γ-secretase generates the Aβ species in two basic product lines from Aβ49 → Aβ46 → Aβ43 to Aβ40 (and Aβ37) and from Aβ48 → Aβ45 → Aβ42 to Aβ38 [5, 6]. Noticeable amounts of Aβ38 are also generated from Aβ43 by a product line switch [7]. Dominantly inherited mutations in the genes encoding PS1 and PS2 manifest with early onset forms of AD (familial AD, FAD) and display a reduced processivity leading to a relative accumulation of the minor Aβ42 and Aβ43 over the major Aβ40 species [8, 9]. The aberrantly increased ratios of Aβ42 or Aβ43, respectively, to Aβ40 are considered to be decisive determinants for the pathogenesis of FAD [10], and can be used for correlations with FAD age of onset. Here, inclusion of short Aβ species as product line end products into Aβ ratios led to even better correlations with age of onset of FAD than the widely used Aβ42/40 ratio [11, 12].

γ-Secretase modulators (GSMs) are small molecule compounds and potential AD therapeutics, which enhance the processivity of γ-secretase such that the generation of short and presumably clinically protective Aβ species such as Aβ38 [13] and/or Aβ37 [11] is increased and that of the pathogenic Aβ42/43 species is lowered [14–16]. Since the seminal discovery of a subset of non-steroidal anti-inflammatory drugs with low potency GSM activity [14], numerous and structural diverse potent compounds with modulatory activities reaching the low nanomolar range have been reported [17]. Some of them have reached further clinical development and testing [18–20]. An important aspect of their mechanism of action is that they, unlike γ-secretase inhibitors, do not affect the total activity of the enzyme by blocking substrate cleavage. As they are expected to leave crucial physiological functions of γ-secretase intact, conceptually, they should be safe Alzheimer drugs that selectively target the generation of pathogenic Aβ forms. Moreover, increasing evidence from in vitro and in vivo studies shows that the shorter species Aβ37, 38 and 40 species inhibit aggregation of the toxic longer Aβ forms [21–24] suggesting an additional beneficial effect of GSMs.

We have previously shown that the activity of most presenilin FAD mutants in generating increased Aβ42 to total Aβ ratios can be inhibited by advanced GSMs [25]. The very aggressive PS1 L166P mutant, which causes an extremely early disease onset in young adulthood [26], is one of the very few mutants which shows GSM resistance in cell culture models including human neuronal cell systems [20, 25, 27–29]. This mutant has been shown to produce rather extreme Aβ42/Aβ40 ratios in cell-based and cell-free γ-secretase assays reaching or even exceeding the amount of Aβ42 over Aβ40 [16, 26, 30].

A certain limitation of most previous studies of presenilin FAD mutants is that their overexpression in cultured cells and transgenic mice causes replacement of the endogenous PS1 and PS2 [31]. While the overexpression models thus mimic a homozygous state, which is advantageous in studying the intrinsic properties of FAD mutants, they do not accurately reflect the genetic state of patients, in which the mutation is almost exclusively affecting only one allele. It thus remains a critical question whether GSM-resistant presenilin FAD mutants such as PS1 L166P would be susceptible to GSMs in a heterozygous state. By addressing this question using heterozygous and homozygous PS1 L166P knock-in (KI) cells, we have found that some, structurally diverse, GSMs can effectively lower the generation of longer Aβ species when the mutant – as in FAD – is present in a heterozygous state. We thus conclude that FAD mutant carriers should positively respond to GSM-treatment even when their mutations generate presenilin proteins that are intrinsically resistant to these compounds.

## Materials and methods

### Antibodies

Antibodies to total Aβ (3552 [32], immunoprecipitation (IP) 1:500, and 2D8 [33], immunoblotting (IB) 1:25 or sandwich-immunoassay (IA) (Meso Scale Discovery (MSD)) 1:1000), PS1 NTF (2G7 [16], IB 3 µg/ml or 2953 [34], IB 1:1000), PS1 CTF (3027 [34], IB 1:1000) and PS2 NTF (2972 [35], IB 1:2000), have been described previously. The anti-PEN-2 antibody 8557 (IB 2 µg/ml) was raised against the N-terminus of PEN-2 (amino acids 4–15, ERVSNEEKLNLC). Antibodies against total Aβ (4G8, BioLegend, Cat. No. 800702, IA 1:1000), NCT (N1660, Sigma, IB 1:10.000 or Nicastrin Polyclonal Antibody, Invitrogen Cat. No. PA1-758, IB 1:1000), PS2 CTF (AD5, Abcam, Cat. No. ab51249, IB 1:1000), poly-histidine-tag (Penta-His, Qiagen, Cat. No. 34660, IB 1:1000), MAP2 (Millipore, Cat. No. AB15452, immunofluorescence (IF) 1:2000), Tau (DA9, ALZFORUM, IF 1:500), β3-Tubulin (Tuj1, Covance, Cat. No. MMS-435P, IF 1:500), Synapsin (Cell Signaling Technology, Cat. No. 5297, IF 1:500), SSEA4 (Abcam, Cat. No. ab16287, IF 1:500), NANOG (Cell Signaling Technology, Cat. No. 4903, IF 1:500), Tra-1–60 (Millipore, Cat. No. MAB4360_2016625, IF 1:500), Oct4 (Stemgent, Cat. No. S090023, IF 1:500) and anti-Aβ37 antibody (D2A6H, Cell Signaling Technology, Cat. No. 12467) as well as anti-mouse/rabbit/rat/chicken Alexa Fluor 488/568/647 (488 mouse: Thermo Fisher, Cat. No. A21121568, 568 rabbit: Thermo Fisher, Cat. No. A11011, 647 rat: Thermo Fisher, Cat. No. A21247, 647 chicken: Thermo Fisher, Cat. No. A21447, IF 1:500 each) were obtained from the indicated companies. C-terminal specific antibodies to Aβ40 (BAP24) and Aβ42 (BAP15) were kindly provided by Manfred Brockhaus (Roche Applied Science). Species-specific anti-Aβ antibodies were SULFO-tagged according to the instructions of the supplier (MSD). The SULFO-tagged antibody against Aβ38 was obtained from MSD.

### Cell lines and cell culture

Immortalised embryonic fibroblast cells from mice expressing human APP and either hetero- or homozygously knocked-in PS1 L166P [36] were generated as follows. For the production of mouse embryonic fibroblasts (MEF), all mice interbred were homozygous *APP* YAC transgenic mice. Primary MEFs were isolated from 14 days (E14) post-conception mouse embryos from the crossing of *APP* YAC × *PSEN1* L166P (-/-) with *APP* YAC × *PSEN1* L166P (-/-) mice to establish WT/WT MEF lines as well as *APP* YAC × *PSEN1* L166P ( +/-) with *APP* YAC × *PSEN1* L166P (+ / +) mice to establish WT/L166P and L166P/L166P MEF lines. The E14 embryo bodies were opened, organs removed, rinsed in PBS, minced with a scalpel and treated for 15 min in 5 ml of 0.25% trypsin solution. The tissue was then triturated 5 times with a 1 ml pipet. Trypsin was inactivated by adding 25 ml of medium (DMEM with 25 mM glucose, 10% FBS, 100 U/ml penicillin, 100 μg/ml streptomycin, 0.25 μg/ml amphotericin B, 6 mM glutamine and 1.5 mM pyruvate). The cell suspension was passed through a cell strainer and centrifuged for 15 min at 200 g. The cell pellet was resuspended in fresh media and plated. After 24 h, media was changed again to eliminate dead cells and debris. To obtain immortalised MEF lines, primary cells at passage 2–3 were transfected (FuGENE6, Promega) with a plasmid containing the SV40 T antigen cDNA. Immortalised cells were selected from individual colonies and maintained in media containing 500 μg/ml of geneticin (G418). Cells were kept at 37 °C with 5% CO_2_ in a humidified incubator. MEFs from passage 10 + were used for all the experiments and cultured in DMEM supplemented with 10% FCS and G418 (200 µg/ml). Human embryonic kidney 293 (HEK293) cells and HEK293/sw cells, which stably express the “Swedish” APP FAD mutation K670M/N671L (APPsw) were cultured as described with or without G418 selection, respectively [37]. H4 cells overexpressing APPsw (H4/sw) and HEK293-based Notch1 reporter cell lines were cultured as described [38].

### Generation of doxycycline (DOX)-inducible Neurogenin 2 (Ngn2)-expressing human induced pluripotent stem cells (iPSCs)

To allow simple differentiation of iPSCs into cortical neurons, we used an Ngn2-based differentiation protocol, based on a cell line with a DOX-inducible Ngn2-transgene positioned into the AAVS1 genomic safe harbor locus of the A18945 iPSC line (ThermoFisher, A18945) that was generated by recombinase-mediated cassette exchange of a master cell line (MCL). For the generation of the MCL, we used the pZ:F3‐CAGGS GPHTK‐F [39] gene targeting vector (Addgene, Cat. No. 12666; kindly provided by Prof. Verfaille (Stem Cell Institute Leuven, KU Leuven)), a ‘landing pad’ containing FRT sites framing a GFP- and hygromycin-resistance/thymidine kinase (GFP-2A-HYG-TK) selection cassette. Two million A18944 iPSCs were transfected with 32 μg of the gene targeting vector and 4 μg of AAVS1 locus specific transcription activator-like effector nucleases (TALEN) plasmids, pTALEN-TD_hAAVS1-1L and pTALEN-TG_hAAVS1-1R in Ingenio electroporation solution (Mirus, Cat. No. MIR 50111) using the Gemini X2 Electroporation System (BTX) with 2 pulses at 65 mV for 20 ms in a 1 mm cuvette (Fisher Scientific, Cat. No. 15437270). Cells expressing GFP-2A-HYG-TK were selected by sorting for GFP and with 50 μg/ml hygromycin B starting 3 days after electroporation. Single-cell clone colonies were picked and analysed by genotyping PCR and qPCR genome integrity was checked by standard trisomy 20 qPCR [40] and molecular karyotyping (performed by Life&Brain GmbH) resulting in the selection of clone MCL-P1C11. Next, the Ngn2 gene was inserted into the vector pZ M2rtTA_CAGG TetON-Sox10 with GFP [41] (Addgene, Cat. No. 115241, kindly provided by Prof. Verfaille (Stem Cell Institute Leuven, KU Leuven, Leuven, Belgium)) by replacing Sox10 using EcoRV/AflII, so that it can be expressed under a DOX-inducible promoter. The purified Ngn2-containing plasmid was transfected into the human master iPSC line together with a flippase-encoding plasmid (pCAG-Flpe-GFP, a gift from Connie Cepko, Addgene, Cat. No. 13788). One selected clone was used for CRISPR/Cas9 editing to introduce the PS1 L166P mutation.

### gRNA selection

The design of guide RNAs (gRNAs) and the general quality control after editing of iPSC cells was performed as described previously [40, 42]. gRNAs binding to exon 6 of *PSEN1* were selected using CRISPOR [43]. The selected gRNA (5´-TAGAGATGATATAATAAGCC-3´) was nearly identical to one used in an earlier study [28].

### iPSC maintenance and generation of PS1 L166P KI cells

iPSC experiments were performed in accordance with all relevant guidelines and regulations. iPSCs were grown in Essential 8 Flex Medium (Thermo Fisher, Cat. No. A2858501) on VTN-coated (Thermo Fisher, Cat. No. A14700) cell culture plates at 37 °C with 5% CO_2_ and split twice a week as small clumps after a 5 min incubation in PBS/EDTA. Cells were electroporated as described previously [42, 44] with the following modifications: instead of plasmids, we used ribonucleoprotein (RNP) complexes. Prior to electroporation, DOX-inducible Ngn2-expressing iPSCs on Geltrex (Thermo Fisher, Cat. No. A1413302)-coated plates, harvested with Accutase (Thermo Fisher, Cat. No. A1110501) and counted. 2.5 × 10^5^ cells/reaction were prepared into tubes and, after centrifugation, resuspended in P3 solution (P3 Primary Cell 4D-Nucleofector® Kit, Lonza). The suspension was added to prepared RNP complexes (5 µg recombinant Cas9 (Integrated DNA Technologies) plus 60 pmol gRNA (Synthego) per reaction). To allow homologous repair, 120 pmol ssODN (ultramer™, Integrated DNA Technologies) complement to the sequence of mutation and protospacer adjacent motif (PAM), which carried the desired PS1 L166P mutation in addition to a silent mutation in the PAM, were added. The preparation was electroporated in a 16-well cuvette. After electroporation, StemFlex Medium (Thermo Fisher, Cat. No. A3349401) including RevitaCell (RVC, Thermo Fisher, Cat. No. A2644501) was added to the cells to allow them to recover. Afterwards, the cells were plated on Geltrex-coated plates in StemFlex/RVC. Cells were grown to approximately 50–70% confluence, harvested with Accutase and plated at very low density on Geltrex with StemFlex/RVC to allow for single-cell clone colonies to grow. The colonies were then picked and analysed by RFLP assay using NsiI (New England Biolabs, Cat. No. R0127) as described previously [44]. Successful hetero- and homozygous editing was confirmed by Sanger sequencing of the selected clones. The top five off-target loci, based on either MIT or CFD scores [43, 45] were checked by PCR amplification and subsequent sequencing. qgPCR and nearby SNP sequencing was performed to exclude unwanted on-target effects [40]. General genome integrity was checked by molecular karyotyping and chromosome 20 qPCR. Pluripotency of the selected clones was checked by staining for pluripotency markers SEAA4, NANOG, Tra-1–60 and Oct4.

### Differentiation of iPSCs into Ngn2-induced cortical neurons

For differentiation into cortical neurons, we used DOX induction of Ngn2 [46] in a two-step protocol [47] with some adaptions. DOX-inducible iPSCs carrying the PS1 L166P mutation either on one or both alleles as well as non-edited cells (WT control) were plated into Geltrex-coated wells (day 0) in neural maintenance medium (NM, Neurobasal, Thermo Fisher, Cat. No. 21103–049) plus DMEM/F12 (Thermo Fisher, Cat. No. 11320–074) (1:1 ratio) including penicillin–streptomycin (Thermo Fisher, Cat. No. 21103–049), B27 (with Vit. A) (Thermo Fisher, Cat. No. 17504–044), NEAA (Thermo Fisher, Cat. No. 11140–050), N-2 supplement (Thermo Fisher, Cat. No. 17502048), insulin (5 μg/ml, Sigma, Cat. No. I0516), 2-mercapto-ethanol (5 mM, Thermo Fisher, Cat. No. 21985–023) supplemented with DOX (Sigma, Cat. No. D9891)). The media was exchanged (NM/DOX) for the next two days. On day three, the cells were harvested with Accutase, counted, resuspended in NM/DOX including ROCK inhibitor (RI, Y27632, Selleckchem, Cat. No. S1049) and plated on dried poly-L-ornithine (Sigma, Cat. No. 27378–49-0) and laminin-coated (Thermo Fisher, Cat. No. 23017015) wells with or without coverslips in NM/DOX. On day 6 and 9 cells were fed with NM/DOX complemented with 300 ng/ml puromycin dihydrochloride (puro, VWR, Cat. No. J593). Afterwards, the medium was switched to NM supplemented with 5 μM 5-fluorouracil (5FU, Sigma, Cat. No. F6627) with half feeds to remove the remaining dividing stem and neuron precursor cells. From day 13, cells were grown in NB (Neurobasal, penicillin–streptomycin) with B27 Plus (Thermo Fisher, Cat. No. A3582801) and 5FU. 5FU was removed in the following feeds. The cells were grown until day 24 before treatment started and analysed at day 27.

### GSMs

GSM-1 [48], RO-02 (RO5434400) [49], RO7019009 [16] and BPN-15606 [50] usage and syntheses have been previously described including references to the relevant patent literature for preparation of GSM-1 (WO2006/043064) or RO-02 (WO2009/103652); GSM-1 (CAS number: 884600–68-4) and BPN-15606 (CAS Number: 1914989–49-3) are also commercially available. The benzene sulfonate (besylate) salt of BPN-15606 was used in all experiments except for H4/sw cells. Synthesis of indole-type GSMs RO5254601, RO5218165, RO5218863 and its photocrosslinkable derivative RO6874585 is described in the Supplementary Information.

### GSM treatments

To test their response to GSMs, control and PS1 L166P KI MEF cells were grown until 70–80% confluence. The culture medium was replaced with fresh medium containing GSMs (GSM-1: 2.5 µM; RO-02: 500 nM; RO7019009: 500 nM; BPN-15606: 360 nM; RO5254601: 2.5 µM) or DMSO as vehicle control. Drug doses (Table S1) were selected from dose–response analyses obtained for endogenous γ-secretase in HEK293/sw cells and were ∼tenfold (lower potency GSMs; GSM-1 and RO5254601; IC_50_ (Aβ42) of ∼180 nM and ∼380 nM, respectively) or ∼30-fold (higher potency GSMs; RO-02, RO7019009, BPN-15606; IC_50_ (Aβ42) of ∼15 nM, 14 nM and 12 nM, respectively) above their Aβ42 IC_50_ and ensured a complete reduction of Aβ42 in the WT condition. Additional experiments were performed with 500 nM BPN-15606. After 24 h, the conditioned medium was collected for Aβ analysis. For dose–response experiments with RO5254601 and BPN-15606, HEK293/sw cells were grown to a confluence of 80% before the medium was changed to fresh medium containing either DMSO vehicle control or drug at the indicated concentrations. The cells were incubated for 16–18 h before Aβ analysis of conditioned medium [16, 25]. Single cell clones of H4/sw cells and HEK293-based Notch1 reporter cell lines were cultured as described [38]. Possible effects of compound treatment to cell viability were recorded using the CytoTox-Glo cell viability assay (Promega) following the instructions of the manufacturer. For the GSM treatment of iPSC-derived cortical neurons, the medium of the cells was removed at day 24 and replaced by NB/B27 Plus containing either 500 nM or 2.5 µM RO70190009, 2.5 µM RO5254601 or DMSO as control. After three days of incubation, the medium was harvested, centrifuged and the supernatant was used for Aβ measurements by immunoassays.

### Protein analysis

For the analysis of protein levels of WT or PS1 L166P KI MEF cells, membrane fractions were prepared as described previously [51], separated on 10–20% Tris-Tricine gels (Novex™ 10–20%, ThermoFisher Scientific, Cat. No. EC66252) and analysed by immunoblotting. Immunoblot analysis of secreted Aβ was performed as described [52]. To analyse GSM dose-responses and determine IC_50_ values for Aβ42 inhibition, secreted Aβ species of HEK293/sw cells were analysed by sandwich immunoassay (MSD) using SULFO-tagged C-terminal specific antibodies to Aβ37, Aβ38, Aβ40 and Aβ42 as described [25] or, in case of H4/sw cells by an Aβ42 AlphaLISA immunoassay kit according to the instructions of the supplier (PerkinElmer) as described [38]. Notch1 cleavage reporter assay was performed as described [38]. GSM effects on secreted Aβ species in conditioned medium of MEF KI cells or iPSC-derived neurons were either quantified by ELISA using end-specific C-terminal antibodies to Aβ38, Aβ40, Aβ42 and Aβ43 obtained from IBL [16, 53] or MSD sandwich-immunoassay (Aβ37, see above). Total Aβ in conditioned medium of iPSC-derived neurons were measured using MSD sandwich-immunoassay using anti-Aβ antibodies 2D8 (biotinylated, capture) and 4G8 (detection) as described previously [54]. Individual Aβ species from MEF KI cells were analysed by immunoblotting using antibody 2D8 after immunoprecipitation with antibody 3552 using Tris-Bicine urea SDS-PAGE [16, 55]. Protein bands from immunoblots were quantified using the LAS-4000 image reader (Fujifilm Life Science) and Multi-Gauge V3.0 software for analysis.

### Cell-free γ-secretase activity assay

To assess γ-secretase activity, CHAPSO-solubilised membrane fractions from MEF cells were incubated with recombinant C100-His_6_ [56] together with DMSO or the γ-secretase inhibitor L-685,458 ( [57], InSolution γ-Secretase Inhibitor X, Sigma-Aldrich, Cat. No. 565771) as previously described [52], followed by separation on 10–20% Tris-Tricine gels and immunoblotting using the penta-His antibody.

### Photoaffinity-labeling experiments

Membrane fractions of HEK293 or MEF cells were incubated with RO6874585 in the presence or absence of other GSMs used as competitor compounds as described previously [49]. In brief, after photoactivation, samples were solubilised using SDS-containing buffer and the crosslinked proteins were pulled down with streptavidin-sepharose. Thereafter, samples were analysed by immunoblotting.

### Statistical analysis

Statistical significance was tested using one-way ANOVA and Dunnett’s multiple comparison test. Corresponding DMSO- and GSM-treated samples were regarded as paired samples.

## Results

### GSMs within particular structural classes can lower pathogenic Aβ42 generation in heterozygous PS1 L166P KI cells

**Fig. 1:**
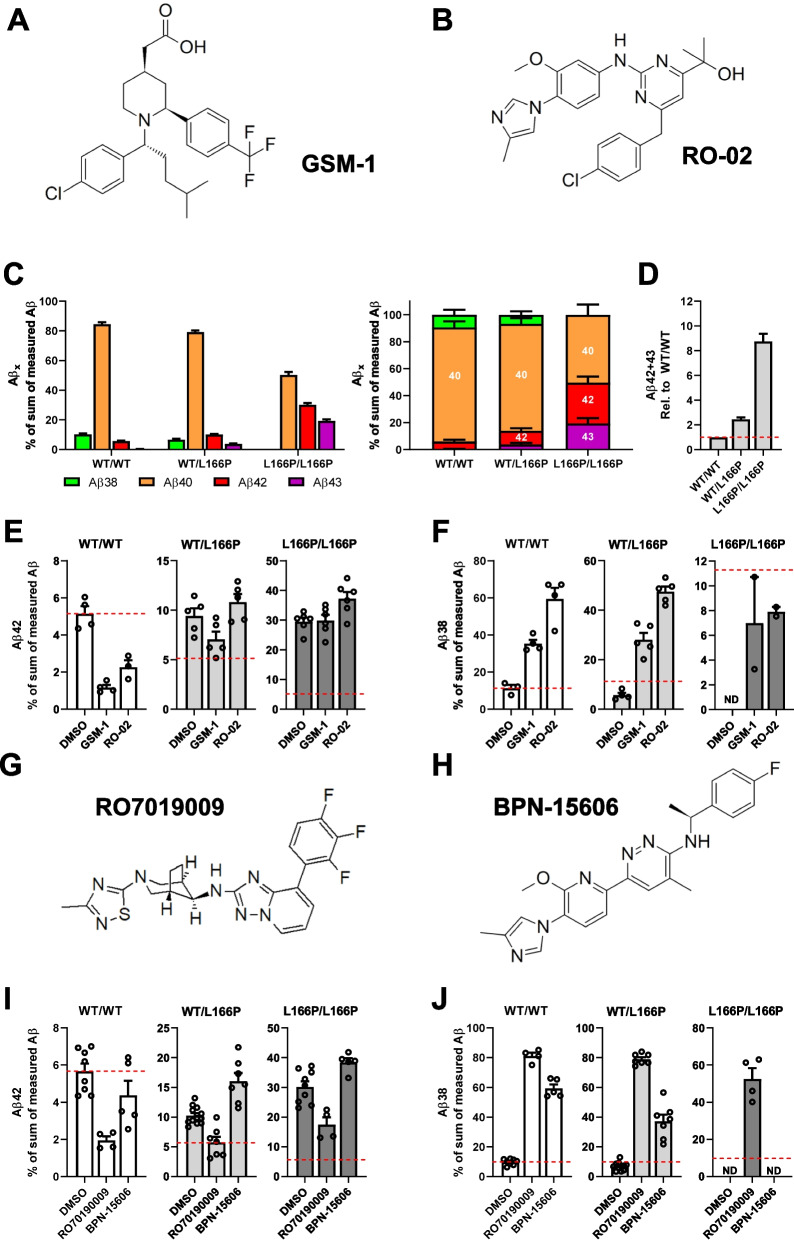
RO7019009 lowers Aβ42 generation in PS1 L166P KI MEF cells. **A** Structure of GSM-1. **B** Structure of RO-02. **C** Ratios of secreted Aβ species (% of the sum of measured Aβ (38 + 40 + 42 + 43)) from untreated MEF KI cells representing the different WT (WT/WT), heterozygous (L166P/WT) or homozygous (L166P/L166P) genotypes (*n* = 13–16). **D** Fold change of the combined ratios of Aβ42 and Aβ43 compared to WT/WT (*n* = 13). **E**, **F** Ratios of secreted Aβ42 (**E**) and Aβ38 (**F**) (% of the sum of measured Aβ (38 + 40 + 42 + 43)) from WT, heterozygous or homozygous KI MEF cells treated with 2.5 µM GSM-1 or 500 nM RO-02 (*n* = 4–6). **G** Structure of RO7019009. **H** Structure of BPN-15606. **I**, **J** Ratios of secreted Aβ42 (**I**) and Aβ38 (**J**) (% of the sum of total measured Aβ (38 + 40 + 42 + 43)) from WT or KI MEF cells treated with 500 nM RO7019009 or 360 nM BPN-15606 (*n* = 4–7). Aβ species were measured by species-specific Aβ ELISA (IBL). Data are shown together with the corresponding DMSO controls and are presented as mean + SEM. The dashed line (**E**, **F**, **I**, **J**) highlights the calculated ratio of the DMSO-treated WT control. Missing data points are due to Aβ levels below the detection limit of the assay. ND; the Aβ species could not be detected in any sample of this condition

So far, we had tested the response of the PS1 L166P mutant to GSMs only under overexpression conditions in transfected cells [16, 25, 48] using prototypic GSMs such as the NSAID-derived acidic compound GSM-1 (Fig. 1A) [48, 58] and RO-02 (Fig. 1B) [49], a non-acidic aromatic bridged heterocyclic GSM, which is more potent than GSM-1 in HEK293 cells stably overexpressing “Swedish” mutant APP (APPsw) harbouring the K670M/N671L FAD mutation (HEK293/sw) [25, 49]. As shown previously [16], despite its much higher potency and activity in increasing the generation of Aβ38, Aβ42 levels generated by PS1 L166P could still not be lowered by this GSM when analysed in cell culture suggesting that the resistance of this mutant is not dependent on compound properties such as basicity or potency. To test whether the GSM resistance to GSM-1 and RO-02 could be broken when PS1 L166P was present at a gene dose of 50% as it is the case in human FAD mutation carriers, where one allele harbors the mutation, we analysed mouse embryonic fibroblast (MEF) cells from a PS1 L166P KI mouse model overexpressing WT human APP [36]. As expected, PS1 NTF and CTF were present in WT (WT/WT) control, heterozygous (WT/L166P) and homozygous (L166P/L166P) KI MEF cells at comparable levels (Fig. S1A), with the reduced γ-secretase activity of PS1 L166P [26] becoming apparent in the homozygous state (Fig. S1). In order to achieve robust modulation effects in these cells, GSMs were used at concentrations according to their potencies (see Materials and Methods). Generation of Aβ was assessed by ELISA using C-terminal end-specific antibodies. As expected, the relative increase of Aβ42 in MEF cells from the WT/L166P KI mice compared to the WT control was much less pronounced as in the homozygous L166P/L166P state (Fig. 1C). Consistent with previous results [16], while Aβ42 was the major pathogenic species produced by PS1 L166P, the mutant also caused a gene-dose dependent increased production of Aβ43, which was barely detectable for WT PS1 (Fig. 1C). Overall, the combined amount of long, pathogenic Aβ42 and Aβ43 was increased by the mutation ~ two-fold in heterozygous and ~ nine-fold in homozygous cells, respectively (Fig. 1D).

As judged from the Aβ42 to Aβ total ratios, heterozygous WT/L166P KI MEF cells were considerably non-responsive to GSM-1 and RO-02 (Fig. 1E, Fig. S2C). However, there was a slight tendency towards weakly reduced Aβ42 ratios after GSM-1 treatment (Fig. 1E). Both compounds caused an efficient increase of Aβ38 in heterozygous KI cells, which was, given the much less pronounced increase in the homozygous cells, likely arising predominantly from the WT allele (Fig. 1F, Fig. S2A). As expected, MEF cells from homozygous PS1 L166P KI mice did not show Aβ42-lowering GSM responses (Fig. 1E). Their GSM resistance was nearly complete as only little if any Aβ38 species were found to be increased (Fig. 1F). Overall, Aβ38 levels in homozygous cells were very low and in case of the untreated cells too low to be measured in the ELISA system. Levels in the treated cells were increased, however, only to about 6–8% of total Aβ (compared with 30–60% in the other genotypes) and still could only be measured in half of the samples.

We next asked whether these small effects on Aβ42 generation in WT/L166P KI MEF cells could be replicated by potent GSMs of different structural classes and if they would be more effective. To this end, we first analysed additional heterocyclic GSMs of high potency and favourable drug-like properties like the piperidine-bridged RO7019009 (Fig. 1G), a compound reaching IC_50_ (Aβ42) values as low as 2 nM in brain-derived cell lines [16] and possessing additional Aβ43-lowering activity [16], as well as BPN-15606 (Fig. 1H), a similarly potent aromatic-bridged heterocyclic GSM ( [50], Fig. S3). As shown in Fig. 1I and Fig. S2C, RO7019009 effectively lowered Aβ42 levels in the heterozygous and also in the homozygous KI cells. In line with these and previous results [16], RO7019009 strongly increased Aβ38 in all different genotypes (Fig. 1J, Fig. S2A). In contrast, BPN-15606 did not lower Aβ42 in either the heterozygous and homozygous conditions (Fig. 1I, Fig. S2C) and had only limited or no effect respectively, on Aβ38 in the mutant cell lines (Fig. 1J, Fig. S2A). WT/WT cells however were modulated by BPN-15606 resulting in reduced Aβ42 levels and including an increase of Aβ38 (Fig. 1I, J, Fig. S2A, C).

**Fig. 2:**
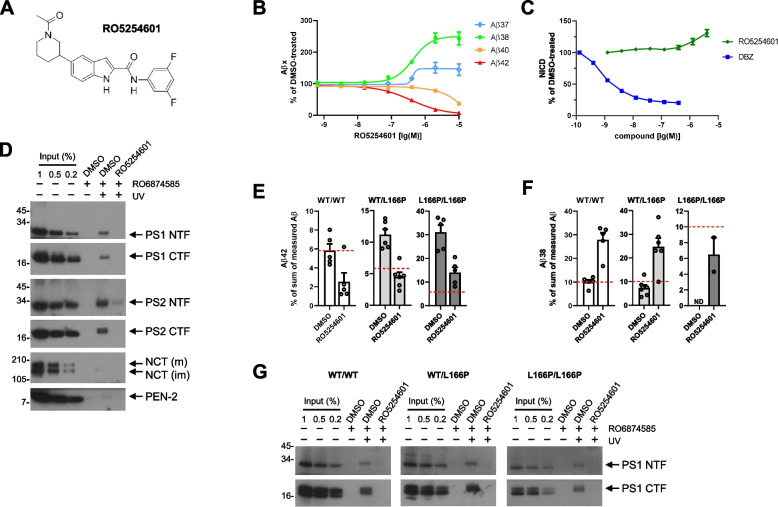
RO5254601, a presenilin NTF and CTF-targeting indole-type GSM reduces Aβ42 in PS1 L166P KI MEF cells. **A** Structure of RO5254601. **B** Dose–response curve of RO5254601 in HEK293/sw cells measured using the MSD sandwich immunoassay (*n* = 5). **C** NICD release measured in a dose–response curve of RO5254601 or the GSI dibenzazepine (DBZ) in a HEK293-based Notch1 reporter cell line (*n* = 4–5). **D** Immunoblot analysis of photoaffinity-labeling experiments of membrane fractions of HEK293 cells with the indole-type GSM RO6874585. Crosslinking (DMSO vehicle control lane) was competed by RO5254601. Samples that were not UV-irradiated were loaded to control for specificity. **E**, **F** Ratios of secreted Aβ42 (**E**) and Aβ38 (**F**) (% of the sum of measured Aβ (38 + 40 + 42 + 43)) WT or KI MEF cells treated with DMSO vehicle or 2.5 µM RO5254601 (*n* = 5–6). The dashed line highlights the calculated ratio of the DMSO-treated WT control. **G** Immunoblot analysis of photoaffinity-labeling experiments of membrane fractions of WT or KI MEF cells with the indole-type GSM RO6874585. Crosslinking (DMSO vehicle control lane) was competed by RO5254601. Samples that were not UV-irradiated were loaded to control for specificity. Aβ species in (**B**) were measured using the MSD sandwich immunoassay and are presented as mean ± SEM. NICD in (**C**) was measured using a reporter assay and are presented as mean ± SEM. Aβ species in (**E**, **F**) were measured by species-specific Aβ ELISA (IBL) and are presented as mean + SEM. Missing data points are due to Aβ levels below the detection limit of the assay. ND; the Aβ species could not be detected in any sample of this condition

We also tested a very different chemical class of potent GSMs based on a novel indole scaffold with subtle differences on the substitution pattern on the aromatic side (acetyl or alkyl piperidine or ether pyrrolidine), also in terms of basicity, for its efficacy to lower Aβ42 generated by the PS1 L166P mutant. RO5254601, an exemplary GSM of this structural class (Fig. 2A) lowered Aβ42 in HEK293/sw cells with an IC_50_ of 383 nM (Fig. 2B). The compound concomitantly increased the production of Aβ38 and, similarly as the acidic GSM-1 [25, 48], left the production of Aβ40 largely unaffected (Fig. 2B). Unlike GSM-1 [25], RO5254601 also increased Aβ37, which is normally less abundant than Aβ38 (Fig. 2B). As expected for a GSM, RO5254601 did not impair Notch1 cleavage, while treatment with DBZ, a prototypic γ-secretase inhibitor (GSI) [59], reduced the levels of the Notch1 intracellular domain (NICD) (Fig. 2C). Structural differences of RO5254601 and in general indole-type compounds compared to known GSMs, prompted us to identify its molecular target in γ-secretase. Photoaffinity-labeling experiments using RO6874585, a biotinylated representative of the indole-type GSMs containing a photoreactive benzophenone group (Fig. S4A), identified the PS1 and PS2 NTF and CTF as specific targets of this compound (Fig. 2D). RO6874585 retained the GSM pharmacology in vitro, lowering Aβ42 in APPsw overexpressing H4 (H4/sw) cells without inhibiting Notch1 processing (Fig. S5A, B). Competition experiments showed that RO5254601 competed with presenilin binding of RO6874585 (Fig. 2D) similarly as its parental compound RO5218863 (Fig. S4B, D). The structurally distinct GSMs RO-02 and GSM-1 did not or only weakly compete binding (Fig. S4D), whereas interestingly, RO5218165 (Fig. S4C), another representative of the indole-type GSMs, and the piperidine-bridged heterocyclic RO7019009 showed differential competition for NTF and CTF binding, respectively (Fig. S4D). Quantification confirmed these observations (Fig. S4E-H). Taken together, the photoaffinity-labeling analysis suggests that the indole-type GSMs bind at the interface of the presenilin heterodimer or alternatively have two different binding sites, one in the presenilin NTF and one in the presenilin CTF. We next tested whether the indole-type GSM RO5254601 was able to lower Aβ42 generated by the PS1 L166P mutant. As shown in Fig. 2E and Fig. S2C, RO5254601 could lower Aβ42 in the heterozygous PS1 L166P state. Strikingly, similar to RO7019009, it could also substantially lower Aβ42 in the homozygous state (Fig. 2E, Fig. S2C). Generation of Aβ38 was efficiently improved in heterozygous compared to homozygous MEF cells (Fig. 2F, Fig. S2A). Finally, photoaffinity-labeling with RO6874585 further demonstrated that PS1 L166P was targeted by indole-type GSMs in the MEF KI cells without apparent differences to the WT (Fig. 2G, Fig. S4I). Taken together, these data show that certain, structurally diverse GSMs can reduce the formation of Aβ42 by PS1 L166P when the mutant is heterozygously expressed as in the natural FAD state.

### Piperidine-bridged RO7019009 can also lower pathogenic Aβ43 generation in heterozygous PS1 L166P KI cells

**Fig. 3:**
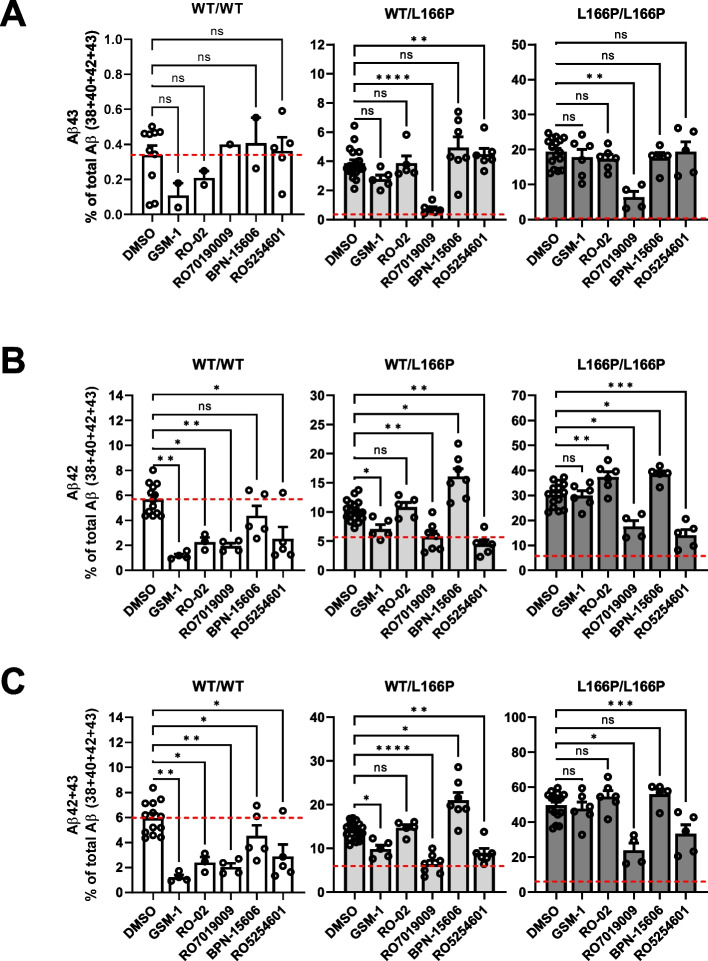
RO7019009 lowers Aβ43 in heterozygous PS1 L166P KI MEF cells. **A**-**C** Ratios of secreted Aβ43 (**A**), Aβ42 (**B**) or Aβ42 + 43 (**C**) (% of the sum of measured Aβ (38 + 40 + 42 + 43)) from WT or KI MEF cells treated with the indicated GSM (GSM-1 and RO5254601 at 2.5 µM, RO-02 and RO7019009 at 500 nM and BPN-15606 at 360 nM, respectively) (*n* = 4–7). Aβ species were measured by species-specific Aβ ELISA (IBL). Data are shown together with the corresponding DMSO controls and are presented as mean + SEM. Dashed red lines highlight the corresponding ratio of the DMSO-vehicle treated WT control. Missing data points are due to Aβ levels below the detection limit of the assay. Statistical significance was tested using one-way ANOVA with Dunnet’s multiple comparison test. Groups with less than two data points were excluded from the analysis

Since PS1 L166P also generates substantial amounts of Aβ43 (Fig. 1C) [16], we next tested whether the levels of this pathogenic Aβ species could be modulated by the above compounds as well. As Aβ43 secretion by WT/WT cells is very low and close to the detection limit of the assay, some of the replicates could not be measured (Fig. 3A, Fig. S2D). Hence, the resulting Aβ43 ratios could be distorted by technical issues and should therefore interpreted with caution. In heterozygous PS1 L166P KI cells, RO7019009 was the only potently effective compound in lowering Aβ43 (Fig. 3A, Fig. S2D). Interestingly, while RO5254601 reduced Aβ42 (Figs. 2E and 3B), it could not lower Aβ43 (Fig. 3A, Fig. S2D), indicating a product line-specific activity. Overall, its robust efficacy to reduce Aβ42 was sufficient to lower the ratio of combined pathogenic species (Aβ42 + Aβ43) similar to RO7019009 (Fig. 3C). These profiles were also observed for homozygous PS1 L166P cells (Fig. 3C). We conclude that the formation of Aβ43 by heterozygous PS1 L166P KI cells can effectively be lowered by RO7019009. This compound as well as the indole-type GSM RO5254601, caused an effective lowering of the generation of total pathogenic Aβ42/43 species generated by PS1 L166P.

### RO7019009 promotes processivity in heterozygous PS1 L166P KI cells in all Aβ product lines

**Fig. 4:**
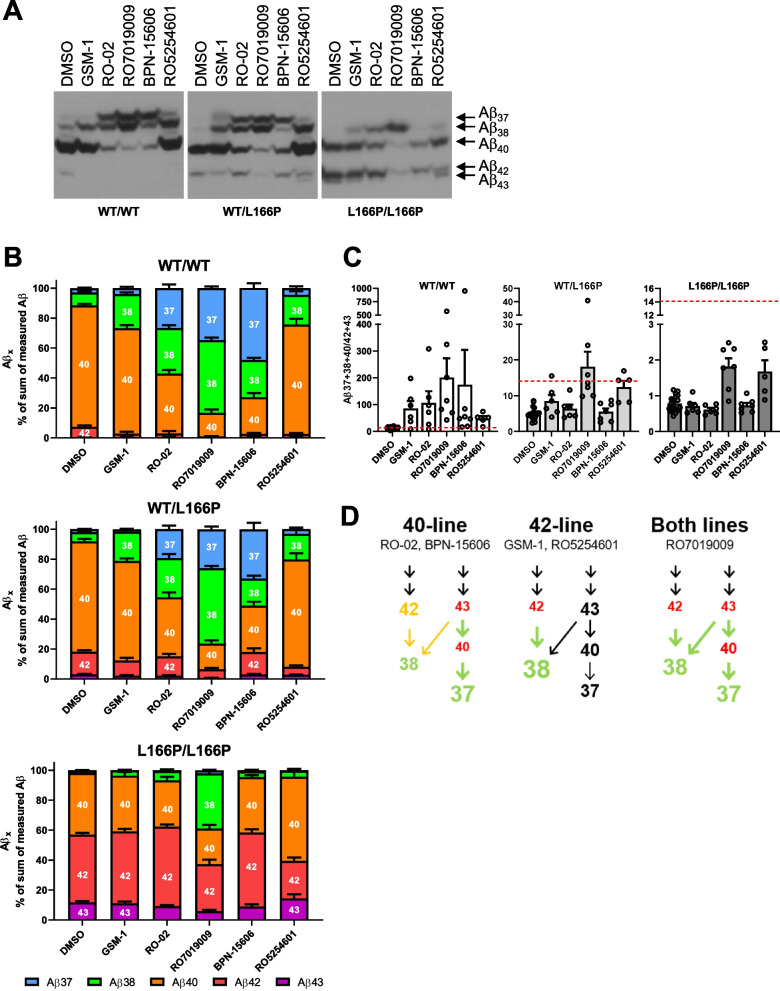
Certain GSMs display Aβ product line specificity. **A** Representative immunoblots of Aβ immunoprecipitated from conditioned medium of WT or KI MEF cells that were treated with the indicated GSMs (GSM-1 and RO5254601 at 2.5 µM, RO-02 and RO7019009 at 500 nM and BPN-15606 at 360 nM, respectively). **B** Ratios of secreted Aβ species (% of the sum of measured Aβ (37 + 38 + 40 + 42 + 43)) from WT or KI MEF cells that were treated with the depicted GSMs (*n* = 5–7). **C** Ratio of the secreted Aβ37 + 38/Aβ42 + 43 representing the processivity in both product lines. WT and KI MEF cells were treated with the depicted GSMs (*n* = 5–7). **D** Schematic overview of the Aβ product lines and their modulation by the various GSMs. Smaller red labelling indicates decreased production of the respective species while bigger green labelling represents increased production. Black and orange coloring indicates unchanged or only little changed Aβ production. Secreted Aβ species (**A**) were separated on Tris-Bicine urea gels and analysed by immunoblotting. The Aβ ratios (**B**, **C**) are shown together with those of the corresponding DMSO controls and presented as mean + SEM. Dashed red lines in (**C**) highlight the corresponding ratio of the DMSO vehicle-treated WT control. Missing data points are due to Aβ signals that could not be quantified

The responses to GSMs are impacted by the L166P mutant in a not yet fully understood manner which may also include Aβ product line switches [8, 16, 48, 60]. To get further insights into how the GSMs affect the processivity of the PS1 L166P mutant γ-secretases, we investigated the impact of the compounds on the Aβ product lines in the WT or heterozygous and homozygous PS1 L166P mutant state in more detail by analysing the full spectrum of Aβ species including also additional shorter species such as Aβ37. Using Tris-Bicine urea SDS-PAGE, which allows separation of the different Aβ species, we assessed differences in the impact of the GSMs on the Aβ product lines. This analysis showed the expected differences between acidic and non-acidic aromatic or piperidine-bridged heterocyclic GSMs in their Aβ modulation behavior (Fig. 4A, B, Fig. S6). In line with previous results [16, 49, 50], and in contrast to GSM-1, high concentrations of the aromatic or piperidine-bridged heterocylic GSMs RO-02, RO7019009 and BPN-15606 caused an increase of both short species, Aβ37 and Aβ38 in WT and heterozygous KI cells (Fig. 4A, B). In addition, these GSMs caused a strong reduction of Aβ40, indicating an increased processivity in the Aβ40 product line (Fig. 4A, B). This product line-preference was further shown for BPN-15606 when it was used at 500 nM, which resulted in a strong reduction of Aβ43, particularly in homozygous cells (Fig. S7). Even though the higher concentrations enhanced the modulatory effect of BPN-15606 its effects were still specific for the Aβ40 product line. Untypical for a non-acidic GSM, the less basic acetyl indole-type GSM RO5254601 behaved very similar to the acidic compound GSM-1, with little effect on Aβ40 and acting essentially only on the Aβ42 product line. RO5254601 effectively acted on this product line also in homozygous KI cells thereby markedly lowering Aβ42 in this genetic state (Fig. 4A, B). In these cells, the processivity-enhancing effect of RO7019009 was largely resulting in an increased generation of Aβ38 (Fig. 4A, B). Analysis of the ratio of shorter Aβ37/38 combined with Aβ40 to longer Aβ42/43 species [12] demonstrated the increased processivity in heterozygous and homozygous KI cells after treatment with piperidine-bridged RO7019009 (Fig. 4C, Fig. S8). These observations corroborate that RO7019009 is the most suitable compound in our study for lowering pathogenic Aβ42/43 species in PS1 L166P KI cells due to its high activity on both product lines. Figure 4D summarises the effects of the GSMs on the final cleavages in the Aβ product lines for the heterozygous PS1 L166P status. The effectiveness in all production pathways to short Aβ species from Aβ42 → Aβ38, Aβ43 → Aβ38, and Aβ43 → Aβ40 followed by Aβ40 → Aβ37 qualifies RO7019009 as an ideal compound to lower the pathogenic Aβ42/43 species produced by the PS1 L166P mutant compared to the other GSMs.

### Pathogenic Aβ42/43 generation can effectively be reduced by RO7019009 in iPSC-derived PS1 L166P KI neurons

**Fig. 5:**
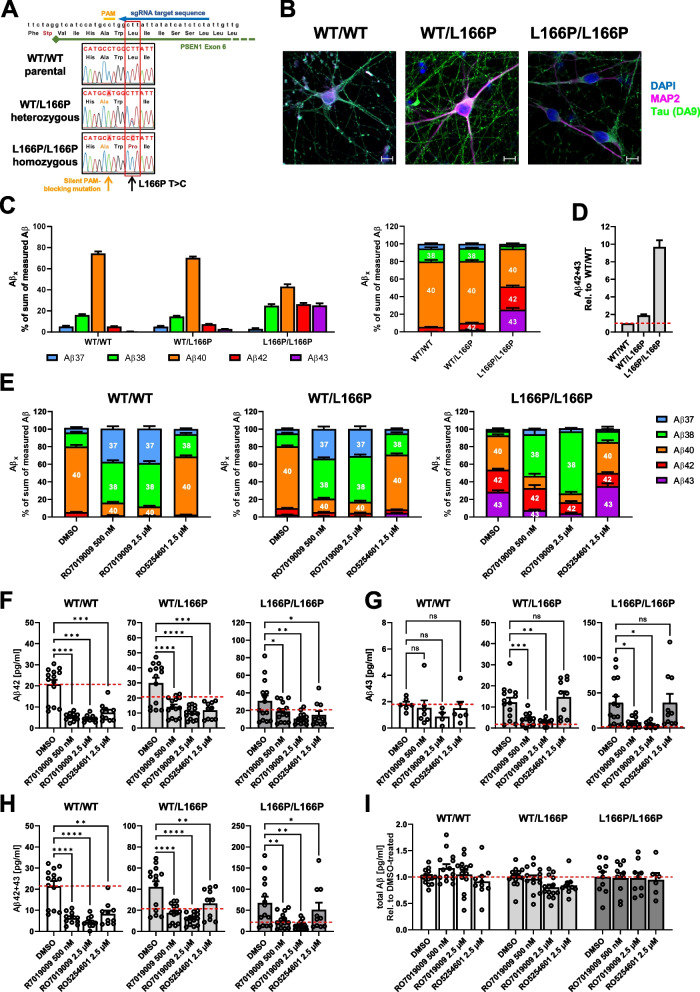
RO7019009 retains its modulatory efficiency in iPSC-derived L166P KI neurons. **A** gRNA binding site in exon 6 of the *PSEN1* gene and Sanger sequencing validation of the inserted edits in hetero- and homozygous PS1 L166P KI cells. Both edited lines (hetero- and homozygous) contain a silent mutation in addition to the desired base exchange at the L166 codon. **B** Representative immunofluorescence stainings of the differentiated neurons at DIV27 (DAPI in blue, MAP2 in pink and tau in green). Single channels are shown in Suppl. Figure 7B; Scalebar: 10 µm. **C** Aβ profiles of parental and edited neurons after DMSO treatment. The different Aβ species are shown as ratios compared to the sum of measured Aβ (Aβ37 + 38 + 40 + 42 + 43) (*n* = 14). **D** Fold change of combined Aβ42 + Aβ43 in the mutant lines compared to parental cells (*n* = 13). **E** Ratios of the measured Aβ species after treatment with 500 nM or 2.5 µM RO7019009, or 2.5 µM RO5254601, compared to the DMSO-vehicle treated control. For reasons of visualization, samples that were below detection limit were set to 0 (*n* = 6–14). **F**–**H** Amount of secreted Aβ42 (**F**), Aβ43 (**G**) or the combined amount of Aβ42 + Aβ43 (**H**) in the supernatant of induced neurons that were treated with GSM or DMSO as control (*n* = 10–14). **I** Total amount of secreted Aβ of the different neuronal cell lines in the presence of DMSO vehicle or GSM. Total Aβ was measured using pan-Aβ antibodies and is displayed relative to DMSO-treated controls (*n* = 10–14). The red dashed line (**F**-**I**) represents the situation in DMSO-treated WT cells. In (**C**-**E**), the Aβ species are displayed as ratio of the sum of measured Aβ (Aβ37 + Aβ38 + Aβ40 + Aβ42 + Aβ43). **F**-**I** shows the amount of measured Aβ species as pg/ml. Missing data points are due to Aβ levels below the detection limit of the assay. Statistical significance (**F**–**H**) was tested using one-way ANOVA with Dunnett’s multiple comparison test

Finally, we tested whether our findings on the effects of the GSMs tested could be translated into human induced pluripotent stem cell (iPSC)-derived cortical neurons, a physiologically more relevant cell model. The *PSEN1 L166P* mutation was edited into the parental iPSCs using CRISPR/Cas9 [40, 61] to obtain heterozygous WT/L166P as well as homozygous L166P/L166P cells (Fig. 5A, Fig. S9). To establish a system with accelerated and simplified neuronal differentiation, we created an iPSC line expressing the transcription factor Neurogenin 2 (Ngn2) upon doxycycline induction [46] (Fig. S10A, for details see Materials and methods and Fig. S11). Ngn2 induction resulted in the rapid formation of neurons that showed typical morphology and expressed neuronal marker proteins such as Tau and MAP2 (Fig. 5B, Fig. S10B). Overall, the Aβ profiles of the differentiated neurons matched those observed in the heterozygous and homozygous MEF PS1 L166P KI cells (Fig. 5C; cf. Figure 1C). Similar to MEF cells, the percentage of longer Aβ42/43 species was increased twofold in heterozygous and ~ tenfold in homozygous L166P/L166P neurons, respectively (Fig. 5D; cf. Figure 1D). This Aβ42/43 increase in heterozygous cells was consistent with previous results [28], while the increase in homozygous cells was slightly stronger than observed previously. Compared to the data obtained from MEF cells, Aβ43 was more strongly increased in the mutant neuronal cells, particularly in the homozygous state. For the analysis of the neuronal cells, the Aβ42/43-lowering piperidine-bridged RO7019009 and the Aβ42-selective indole-type RO5254601 were selected as most interesting compounds. Treatment of the neuronal cells with RO7019009 or RO5254601 resulted in the modulation of γ-secretase processivity (Fig. 5E-H, Fig. S12) without affecting total Aβ levels (Fig. 5I). The effects of the GSMs on WT neurons confirmed the effects in MEF cells. RO7019009 reduced Aβ40 and Aβ42 while increasing both shorter species Aβ37 and Aβ38, whereas RO5254601 primarily acted on Aβ42 and Aβ38 (Fig. 5E). Heterozygous neurons were efficiently modulated by RO7019009, which reduced both Aβ42 and Aβ43 (Fig. 5E-H). In contrast, RO5254601 reduced only Aβ42 and had no effect on Aβ43 (Fig. 5E-H). In homozygous neurons, the overall fraction of longer species was not reduced by RO5254601 (Fig. 5E). RO7019009, however, efficiently reduced not only Aβ40 but also Aβ43 and, especially at higher concentrations, Aβ42 (Fig. 5E-H). While RO7019009 reduced the long Aβ species similarly as in MEF cells, the indole-type GSM RO5254601 barely reduced the sum of long Aβ42 and Aβ43 species in the homozygous neurons (Fig. 5E, H, cf. Figure 3C), which can be explained by the poor effect of RO5254601 on the Aβ40 product line and the higher ratios of Aβ43 in the neurons. Taken together, RO7019009 most effectively lowered both pathogenic Aβ42/43 species in iPSC-derived neuronal cells in the heterozygous and to a lesser extent in the homozygous PS1 L166P KI state.

## Discussion

PS1 L166P is one of the most aggressive presenilin FAD mutants known to date [26]. In this study, we have shown that some GSMs can break the intrinsic GSM resistance of this mutant when it is expressed heterozygously as in the case of individuals carrying the mutation. However, there were some differences between the KI cell models utilised. The Aβ profile was similar in MEF and human neuronal cells with a two-fold increase of pathogenic Aβ42/43 species in heterozygous and up to ten-fold increase in homozygous cells, respectively. In the homozygous neuronal L166P/L166P KI cells, however, relative to Aβ42, Aβ43 was more prominently increased compared to the corresponding MEF KI cells. In MEF cells that were derived from heterozygous PS1 L166P KI-mice, Aβ profiles were modulated by several GSMs. In contrast, homozygous cells were overall largely GSM-resistant, reflecting the effects of “homozygous” overexpression in HEK293 cells [16, 25, 48], and were only modulated by RO7019009 and RO5254601. The CRISPR/Cas9-edited iPSC-derived neurons could be modulated by RO7019009 also in both heterozygous and homozygous genotypes with similar effects on the Aβ production. In contrast, the indole-type GSM RO5254601 was less effective, also in heterozygous cells, probably to the higher amount of Aβ43 in the human model system. We note that previous studies with iPSC-derived neuronal cells from a small number of *PSEN1* FAD mutation carriers showed beneficial Aβ42-lowering responses to the treatment with non-acidic GSMs [62, 63]. In addition, cell-free γ-secretase assays using PS1 FAD brain samples to analyse the effect of non-acidic and acidic GSMs on Aβ38/42 processivity ratios as a readout indicated that these compounds could be effective and attenuate the increased Aβ42/40 ratios caused by the PS1 FAD mutants [64]. However, as far as investigated, the PS1 FAD mutants analysed in the above studies are per se responsive to GSMs in presenilin overexpression models [20]. In addition, these mutants are rather modest with respect to their pathogenicity and have a much later disease onset than the very aggressive PS1 L166P mutant analysed here. As exemplified for this mutant, our study now suggests that FAD mutations in presenilin previously thought to be GSM-resistant (for an overview see [20]) can be in principle sensitive to modulation by GSMs even when data obtained in homozygous or overexpression cell lines might suggest the opposite. This suggests that potential future treatments with clinically suitable GSMs should be effective in lowering pathogenic Aβ species for carriers of such presenilin mutations. Yet, careful assessment of the GSM pharmacology in terms of potency and effects on Aβ profiles will be essential for each specific presenilin mutation for a clinical application.

Our study also provides further insights on the mechanism of how FAD mutations respond to GSMs. A complex picture showing individual responses to GSMs depending on mutation and compound has emerged in previous studies (reviewed in [20]). While most of these studies used overexpression models [16, 25, 27, 48, 65] allowing to study the intrinsic activities of presenilin FAD mutants, the situation is more complex in FAD. Here, the remaining WT *PSEN1* allele and the two WT *PSEN2* alleles represent 75% of the presenilin gene dosage, so that the observed γ-secretase activity largely comes from the WT presenilins. Indeed, and remarkably, the heterozygous state in both KI cell models used in this study showed an unexpectedly low increase in longer, pathogenic species, which, compared to homozygous cells, did not reflect the mutant gene-dosage. This finding is consistent with previous studies [28, 36] but differs from a corresponding analysis of PS1 M146V, for which the Aβ ratio approximately doubled in a gene dose-dependent manner [28], as would be expected. The only slight increase in the Aβ ratio observed in the presence of one healthy PS1 allele may be attributed to the severe loss of function of PS1 L166P [26], which manifested in the homozygous cells. As a consequence, the reduced activity of the mutant allele is overridden in the heterozygous cells by that of the remaining WT PS1 copy, which produces Aβ40 as the main Aβ species. In the homozygous situation, however, where Aβ42 and Aβ43 dominate over Aβ40, the levels of these rise to an excessive degree. Surprisingly, the relatively subtle increase of longer species in heterozygous cells appears to be sufficient to cause the drastic clinical phenotype with an unusual early age of onset of the disease in *PSEN1 L166P* FAD individuals [26].

In contrast to overexpression models, the response to a given GSM is a composite of the drug responses of the WT and mutant proteins present in the KI cells. Consequently, for a mutant such as PS1 L166P, which is intrinsically largely refractory to GSMs, the net drug response may be reduced and even masked by that of the WT protein. However, the RO7019009-mediated reduction of Aβ43, which is barely produced by the WT allele, demonstrated the contribution of the mutant allele to the drug response in the patient-relevant heterozygous situation.

In the PS1 L166P KI cell culture models studied here, the GSMs acted differentially on the Aβ product lines resulting in distinct net responses to the mixed presenilin genotype. For example, RO-02 and BPN-15606 strongly increased the production of shorter species in heterozygous MEF KI cells without the expected concomitant strong lowering of Aβ42 and Aβ43. Therefore, it seems that the increased production of shorter species is primarily caused by increased cleavage of Aβ40 (i.e., Aβ40 → Aβ37) and/or its precursor Aβ43 (likely including Aβ43 → Aβ38 cleavage for RO-02). The efficient lowering of Aβ42 for other PS1 FAD mutants, however, such as PS1 A246E or PS1 ∆exon9 by BPN-15606 [50, 66, 67] indicates a mechanistically interesting, not yet understood effect of the PS1 L166P mutation on BPN-15606 activity. In contrast to RO-02 and BPN-15606, RO7019009 efficiently reduces Aβ42 and Aβ43. Thus, RO7019009 is the most efficient GSM compound against the pathogenic longer Aβ species. In addition, RO7019009 is a strong activator of processivity as shown by the increased ratio of the shorter products Aβ37, Aβ38 and Aβ40 to their direct and indirect precursors Aβ42/Aβ43 (Aβ37 + 38 + 40/Aβ42 + 43), compared to the other two aromatic-bridged heterocyclics RO-02 and BPN-15606, which also increase both short species, but do so less effectively. Interestingly, RO7019009 was more effective in lowering Aβ42 in the PS1 L166P KI cell models than in the overexpression cell model [16] suggesting that KI cells with endogenous expression may offer a potential advantage over overexpression models for drug-response analysis.

The herein characterised novel indole-type GSM RO5254601 caused a reduction of Aβ42 levels and increased secretion of Aβ38, while Aβ40 and Aβ43 levels were largely unchanged. This resembles the mode of action of classic acidic GSMs like GSM-1, which act primarily on the Aβ42 line and cause increased cleavage of Aβ42 to Aβ38. However, unlike GSM-1, the structurally different indole-type GSM also increased the processivity in the Aβ42 line in homozygous cells, leading to a reduction of Aβ42. This identifies RO5254601 as GSM with exceptional behavior, which can break the intrinsic resistance of this mutant towards lowering of Aβ42 by selectively modulating the Aβ42 product line, while leaving the Aβ40 product line unaffected. Photoaffinity-labeling experiments showed that the GSMs of this class target the presenilin NTF and CTF suggesting that they bind at the NTF/CTF interface, or have separate binding sites in the two subunits. Such affinity-labeling patterns have to our knowledge not been observed for other GSMs, suggesting that the indole-type GSMs target one or more sites in γ-secretase; likely largely distinct from the binding site of other GSMs, all of which have been shown to target the presenilin NTF [49, 68–71]. This interpretation was also supported by the labeling-competition experiments with the structurally different GSMs used in this study.

Interestingly, the two most effective GSMs, RO5254601 and RO7019009 seem to work slightly differently. While RO5254601 reduces the levels of Aβ42 almost exclusively, RO7019009 reduces Aβ43 more efficiently than Aβ42. As known from acidic GSMs, the indole-type GSM RO5254601 has nearly no effect on the main species Aβ40, while Aβ40 is strongly affected by RO7019009. This difference is also reflected by the distinct effect of these two GSMs with respect to the different Aβ ratios that could be used to visualise processivity. The choice of Aβ species being compared in ratios strongly influences the observed increases in processivity. If Aβ40 is included in the analysis (e.g., in the Aβ37 + 38 + 40/Aβ42 + 43 ratio), both RO7019009 as well as RO5254601 boost γ-secretase processivity, while RO5254601 seems to be hardly effective if only short and long Aβ species are compared. This highlights the importance of careful selection of the analysed Aβ species to avoid missing effects of GSMs that act via slightly distinct mechanisms.

The fact that both modulators potently modulate heterozygous PS1 L166P KI cells indicates that the preference for one of the two product lines is less important for overall GSM efficiency. The remarkable ability of RO7019009 to act on both product lines however can explain its exceptional efficiency to reduce longer Aβ species in both hetero- and homozygous MEF and neuronal cell systems. The changed efficiency of RO5254601 in MEF and neuronal heterozygous cells that most probably is due to the slight changes in the production of Aβ42 and Aβ43 highlights the strong influence of the careful choice of a GSM dependent on the specific situation. These data indicate that the modulatory activity of GSMs not only depends on their own characteristics and/or structural class but is also influenced by the particular γ-secretase complex that is targeted and its potential mutational status.

## Conclusions

In summary, our data indicate that heterozygous carriers of intrinsically largely resistant FAD mutants should be susceptible to the positive effects of GSM treatment in lowering Aβ42 and Aβ43 species. Since short Aβ species have been shown to cause reduced aggregation of Aβ42 in heterogenous Aβ mixtures as they occur in the brain [24] and prevent Aβ42 toxicity in in vivo models [22, 23], increasing their abundance by GSM treatment could cause beneficial effects. Remarkably, increased CSF levels of Aβ38 were recently found to correlate with better cognitive performance and less cognitive decline in two pre-AD clinical cohorts [13]. Moreover, the CSF Aβ37/42 ratio has been shown to be a better biomarker for AD than the canonical Aβ42/40 ratio [11]. Our data support the use of clinically suitable GSMs as a safe and preferred strategy to lower pathogenic Aβ forms to prevent AD in both its sporadic and familial forms.

## Supplementary Information

Supplementary Material 1: Figure S1. γ-Secretase activity in WT and PS1 L166P KI MEF cells. (A) Immunoblot analysis of γ-secretase subunits (left panel) and APP processing (right panel) in membrane fractions of WT or PS1 L166P KI MEF cells. (B) Quantification of the expression levels of PEN-2 from (A) (n = 6) (C) Quantification of the relative AICD generation compared to the total levels of product and educts (AICD+APP CTFs) (n = 6). (D) Representative immunoblot analysis of γ-secretase activity (AICD generation) in membrane fractions of WT or PS1 L166P KI MEF cells using a cell-free C100-His_6_ cleavage assay. Values in (B) and (C) are shown relative to the corresponding WT MEF cell levels. Protein levels in (B) were normalised to calnexin as loading control. Statistical significance (B, C) was tested using one-way ANOVA with Dunnett’s multiple comparison test.

Supplementary Material 2: Figure S2. Concentrations of Aβ species secreted by WT or PS1 L166P KI MEF cells treated with different GSMs. (A-E) Measured amounts (pg/ml) of Aβ38 (A), Aβ40 (B), Aβ42 (C), Aβ43 (D) and Aβ42+43 (E) in the medium of WT (WT/WT) and heterozygous (WT/L166P) or homozygous (L166P/L166P) KI MEF cells that were treated with 2.5 µM GSM-1, 500 nM RO-02, 500 nM RO7019009, 360 nM BPN-15606 or 2.5 µM RO5254601 (n = 4–7). Aβ species were measured by species-specific Aβ ELISA (IBL). Data are shown together with the corresponding DMSO controls and are presented as mean + SEM. Dashed red lines highlight the corresponding ratio of the DMSO-treated WT control. Missing data points are due to Aβ levels below the detection limit of the assay. ND; the Aβ species could not be detected in any sample of this condition.

Supplementary Material 3: Figure S3. Dose-response analysis of BPN-15606. Dose-response curves of BPN15606 in HEK293/sw cells measured using the MSD sandwich immunoassay (n = 4–6). In the lower panel, Aβ37 and Aβ38 were excluded to enlarge the effects on Aβ40 and Aβ42. Data are presented as mean ± SEM.

Supplementary Material 4: Figure S4. Indole-type GSMs bind to the presenilin NTF and CTF. (A) Structure of the photocrosslinkable compound RO6874585. Compound moieties are colored in blue (GSM), red (benzophenone) and orange (biotin). (B) Structure of RO5218863, the parental compound of the crosslinkable derivate RO6874585. (C) Structure of the additional indole-type GSM RO5218165. (D) Immunoblot analysis of photoaffinity-labeling experiments with the indole-type GSM RO6874585. Competition of crosslinking (DMSO control) was analysed using a 100x excess of RO5254601, the parental compound RO5218863, RO-02, GSM-1, RO5218165 or RO7019009. Samples that were not UV-irradiated were loaded to control for specificity. (E-H) Quantification of crosslinking efficiencies of PS1 NTF (E), PS2 NTF (F), PS1 CTF (G) and PS2 CTF (H) in HEK293 cell membranes in the presence of the indicated GSM (n = 4–6).(I) Quantification of crosslinking efficiencies of PS1 NTF and PS1 CTF in WT or PS1 L166P KI MEF cell membranes (n = 4–5). Data in (E-I) are presented as mean + SEM. Dashed red lines (E-H) highlight the crosslinking efficiency of the DMSO vehicle-treated control.

Supplementary Material 5: Figure S5. Potencies of the GSMs used. (A) The Aβ42-lowering effects of the GSMs used in this study were additionally characterised in dose-response curves on the H4/sw cell line, measuring Aβ42 levels after over-night incubations with an Aβ42-AlphaLisa. Effects of compounds on Notch1 processing were recorded using a Notch1 reporter assay. Effects of compounds on cell viability were recorded using the same compound concentrations and incubation conditions as in the Aβ42 and Notch1 assays, respectively. The GSI DBZ was included as reference. Means ± SEM of 4–7 independent experiments with 2 technical replicates each are shown. (B) IC_50_ values for Aβ42 calculated from the experiments in (A).

Supplementary Material 6: Figure S6. Ratios of Aβ species secreted by WT or PS1 L166P KI MEF cells as analysed by Tris-Bicine urea SDS-PAGE. (A-F) Ratios of Aβ37 (A), Aβ38 (B), Aβ40 (C), Aβ42 (D), Aβ43 (E) and combined ratio of Aβ42+43 (F) expressed as % of the sum of measured Aβ (37+38+40+42+43) from WT (WT/WT) and heterozygous (L166P/WT) or homozygous (L166P/L166P) KI MEF cells treated with the depicted GSMs (GSM-1 and RO5254601 at 2.5 µM, RO-02 and RO7019009 at 500 nM and BPN-15606 at 360 nM, respectively) (n = 5–7). Aβ species were separated on Tris-Bicine urea gels and analysed by immunoblotting. The calculated ratios are shown together with those of the corresponding DMSO controls and presented as mean + SEM. Dashed red lines highlights the corresponding ratio of the DMSO vehicle-treated WT control. Missing data points are due to Aβ signals that could not be quantified.

Supplementary Material 7: Figure S7. Effect of an increased dose of BPN-15606 on the secretion of Aβ in WT and PS1 L166P KI MEF cells. (A-C) Ratios of Aβ40 (A), Aβ42 (B) and Aβ43 (C) expressed as % of the sum of measured Aβ (37+38+40+42+43) from WT (WT/WT) and heterozygous (L166P/WT) or homozygous (L166P/L166P) KI MEF cells treated with DMSO or 500 nM BPN-15606 (n = 10). Aβ species were separated on Tris-Bicine urea gels and analysed by immunoblotting. The calculated ratios are presented as box-whiskers-plot.

Supplementary Material 8: Figure S8. Ratios between different Aβ species. (A) Ratio of the short Aβ37/Aβ38 species representing direct or indirect products of the longer pathogenic Aβ42/Aβ43 species (n = 5–7). (B) Ratio of Aβ37 to Aβ42 (n = 5–7). (C) Ratio of Aβ42 to Aβ40 (n = 5–7). Aβ species were separated on Tris-Bicine urea gels and analysed by immunoblotting. The values in (A-C) were calculated from the ratios of the single Aβ species. Data are shown together with the corresponding DMSO controls and presented as mean + SEM. The red dashed line highlights the corresponding ratio of the DMSO vehicle-treated WT control. 

Supplementary Material 9: Figure S9. Quality control and validation of edited Ngn2-inducible iPSCs containing one or two PS1 L166P alleles. (A) List of predicted most likely off-target sites of the used gRNA. None of the sites showed any alteration after editing. (B, C) Analysis of CRISPR-mediated on-target effects by qgPCR quantitation of allele copy number (B) and Sanger sequencing of SNPs near the edited locus in WT and PS1 L166P iPSC lines (C) shows maintenance of both alleles after editing. (D) Single channel and merged immunofluorescence stainings of pluripotency markers Tra-1-60, Oct4, SSEA4, and NANOG in edited iPSCs. Scalebar: 50 µm.

Supplementary Material 10: Figure S10. Ngn2-expressing iPSCs can be differentiated into cortical neurons after doxycycline induction. (A) Schematic workflow of the differentiation protocol (DOX, doxycycline; Puro, puromycine; 5FU, 5-fluorouracil), see Materials and methods for details. (B) Single channel and merged immunofluorescence stainings (DAPI (blue), Tau (green), MAP2 (pink) and Tuj1 (green)) of differentiated neurons at day 27. The merged picture is also shown in Fig. 5B. Magnification: 63x; Scalebar: 10 µm.

Supplementary Material 11: Figure S11. Quality control and validation of the flippable master cell line (MCL) iPSCs. (A, B) PCR-genotyping (A) of correct integration of the GFP-HYG-TK cassette in the AAVS1 locus of the MCL candidates in the selected cell clones. The B6 cell line was used to control the primer specificity for the cassette (A). MCLs were characterised as homozygous (Homo) or heterozygous (Hetero) based on the presence of a WT band or the amplification product of the 5′/3′ junction regions of the cassette (A), which was further confirmed by the relative GFP-HYG-TK cassette copy number (B). (C) Clones were screened for genetic alterations in the anti-apoptotic gene BCL2L1. (D) Representative immunohistochemistry image of a MCL clone expressing the pluripotency markers Tra-1-60 (cytoplasmic) and Oct4 (nuclear). Scale bar 100 μm. (E) Representative immunocytochemistry images of GFP expression in undifferentiated MCL cells (top) or after recombinase-mediated cassette exchange by which the GFP-HYG-TK cassette of the MCL is exchanged by the DOX-induced transcription factors resulting in a loss of GFP expression (bottom). Data in (B) are represented as mean ± SD of 2 independent experiments. Data in (C) are represented as the mean of 2 technical replicates.

Supplementary Material 12: Figure S12. Levels of Aβ secreted by iPSC-derived neurons. (A-C) Measured amounts (pg/ml) of Aβ37 (A), Aβ38 (B) and Aβ40 (C) in the medium of WT (WT/WT), heterozygous (WT/L166P) or homozygous (L166P/L166P) PS1 L166P KI neurons that were treated with DMSO, RO7019009 (500 nM and 2.5 µM) or RO5254601 (2.5 µM) (n = 10–14). Aβ species were measured by species-specific Aβ ELISA (IBL) (Aβ38 and Aβ40) or electrochemiluminescence immunoassay (MSD, Aβ37) and are presented as mean + SEM. The dashed line highlights the levels of the DMSO vehicle-treated WT control. Missing data points are due to Aβ levels below the detection limit of the assay.

Supplementary Material 13: Table S1. Overview of IC_50_ (Aβ42) values and GSM concentrations tested.

Supplementary Material 14. Supplementary Information. Indole-type GSMs.

## Data Availability

No datasets were generated or analysed during the current study.

## Abbreviations

AD: Alzheimer´s disease
APP: β-Amyloid precursor protein
APPsw: “Swedish” mutant APP
Aβ: Amyloid-β peptide
CTF: C-terminal fragment
DBZ: Dibenzazepine
DOX: Doxycycline
FAD: Familial AD
5FU: 5-Fluorouracil
gRNA: Guide RNA
GSI: γ-Secretase inhibitor
GSM: γ-Secretase modulator
H4/sw: H4 cells stably overexpressing APPsw
HEK293/sw: HEK293 cells stably overexpressing APPsw
HEK293: Human embryonic kidney 293
IA: Sandwich-immunoassay
IB: Immunoblotting
IC_50_: Concentration of a drug for 50% inhibition
IF: Immunofluorescence
IP: Immunoprecipitation
iPSC: Induced pluripotent stem cell
KI: Knock-in
MCL: Master cell line
MEF: Mouse embryonic fibroblast
MSD: Meso Scale Discovery
NB: Neurobasal
Ngn2: Neurogenin 2
NICD: Notch1 intracellular domain
NM: Neural maintenance medium
PAM: Protospacer adjacent motif
PS1: Presenilin-1
RI: ROCK inhibitor
RNP: Ribonucleoprotein
RVC: RevitaCell
TALEN: Transcription activator-like effector nucleases
