# Correction: γ‑Secretase modulator resistance of an aggressive Alzheimer‑causing presenilin mutant can be overcome in the heterozygous patient state by a set of advanced compounds

**DOI:** 10.1186/s13195-025-01723-9

**Published:** 2025-04-15

**Authors:** Johannes Trambauer, Rosa Maria Rodriguez Sarmiento, Holly J. Garringer, Katja Salbaum, Liliana D. Pedro, Dennis Crusius, Ruben Vidal, Bernardino Ghetti, Dominik Paquet, Karlheinz Baumann, Lothar Lindemann, Harald Steiner

**Affiliations:** 1 Division of Metabolic Biochemistry, Faculty of Medicine, Biomedical Center (BMC), LMU Munich, Feodor‑Lynen‑Str. 17, Munich, 81377 Germany; 2 German Center for Neurodegenerative Diseases (DZNE), Munich, 81377 Germany; 3 Pharma Research and Early Development, F. Hoffmann-La Roche AG, Therapeutic Modalities, Small Molecule Research, Roche Innovation Center Basel, Basel, 4070 Switzerland; 4 Department of Pathology and Laboratory Medicine, Indiana University School of Medicine, Indianapolis, IN 46202 USA; 5 Institute for Stroke and Dementia Research, University Hospital, LMU Munich, Munich, 81377 Germany; 6 Munich Cluster of Systems Neurology (SyNergy), Munich, 81377 Germany; 7 Pharma Research and Early Development, F. Hoffmann-La Roche AG, Neuroscience and Rare Diseases Translational Area, Neuroscience Discovery, Roche Innovation Center Basel, Basel, 4070 Switzerland

**Correction: Alz Res Therapy 17, 49 (2025)**

**https://doi.org/10.1186/s13195-025-01680-3**

Following the publication of the original article [1], the e-files in the Supplementary Information section were out of sync resulting in the missing Supplementary material 14 e-file. The missing Supplementary material 14 file is given here.

The original article [1] has been updated.

## Supplementary Information

Supplementary Material 14. Supplementary Information. Indole-type GSMs.
