# Supplementary figures and images for: γ-Secretase modulator resistance of an aggressive Alzheimer-causing presenilin mutant can be overcome in the heterozygous patient state by a set of advanced compounds
Source: Alzheimers Res Ther. 2025 Feb 19;17:49. doi: 10.1186/s13195-025-01680-3 (PMC11837686; doi:10.1186/s13195-025-01680-3)

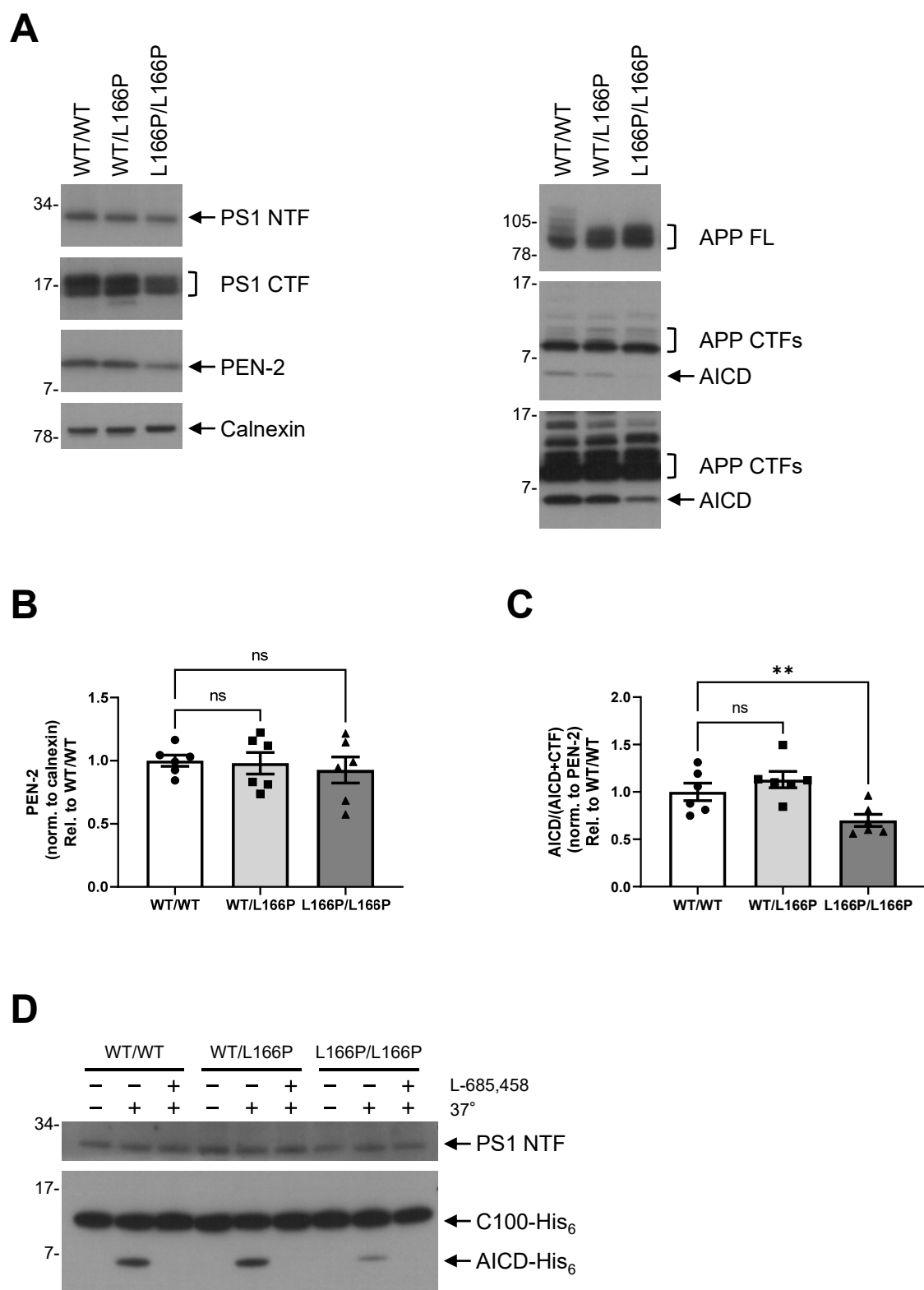

Supplement: Supplementary file 1 — Supplementary Material 1: Figure S1. γ-Secretase activity in WT and PS1 L166P KI MEF cells. (A) Immunoblot analysis of γ-secretase subunits (left panel) and APP processing (right panel) in membrane fractions of WT or PS1 L166P KI MEF cells. (B) Quantification of the expression levels of PEN-2 from (A) (n = 6) (C) Quantification of the relative AICD generation compared to the total levels of product and educts (AICD+APP CTFs) (n = 6). (D) Representative immunoblot analysis of γ-secretase activity (AICD generation) in membrane fractions of WT or PS1 L166P KI MEF cells using a cell-free C100-His6 cleavage assay. Values in (B) and (C) are shown relative to the corresponding WT MEF cell levels. Protein levels in (B) were normalised to calnexin as loading control. Statistical significance (B, C) was tested using one-way ANOVA with Dunnett’s multiple comparison test. [file 13195_2025_1680_MOESM1_ESM.pdf]

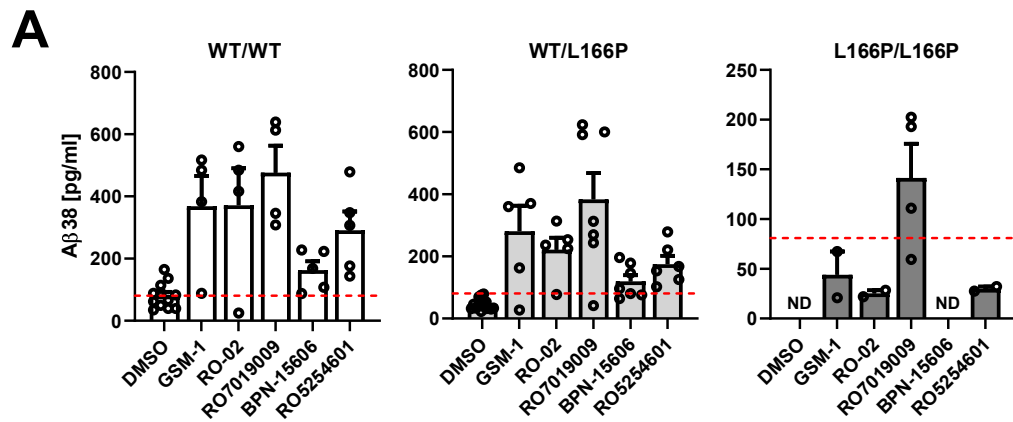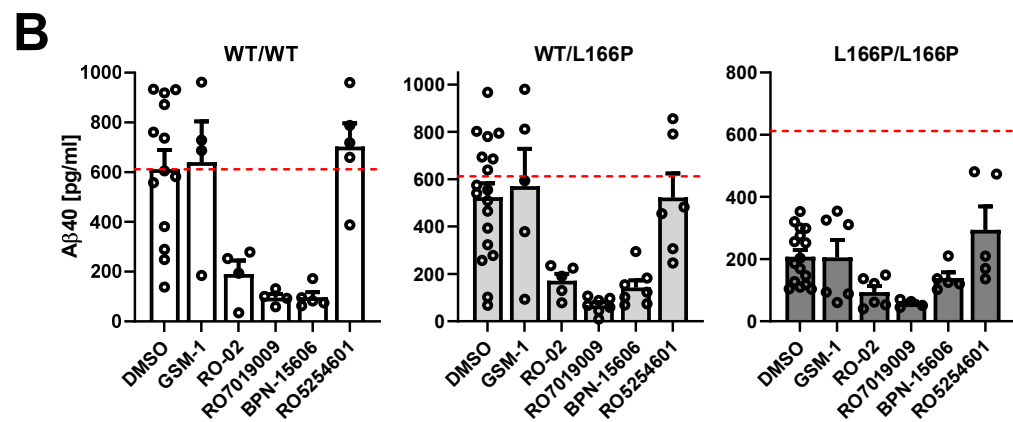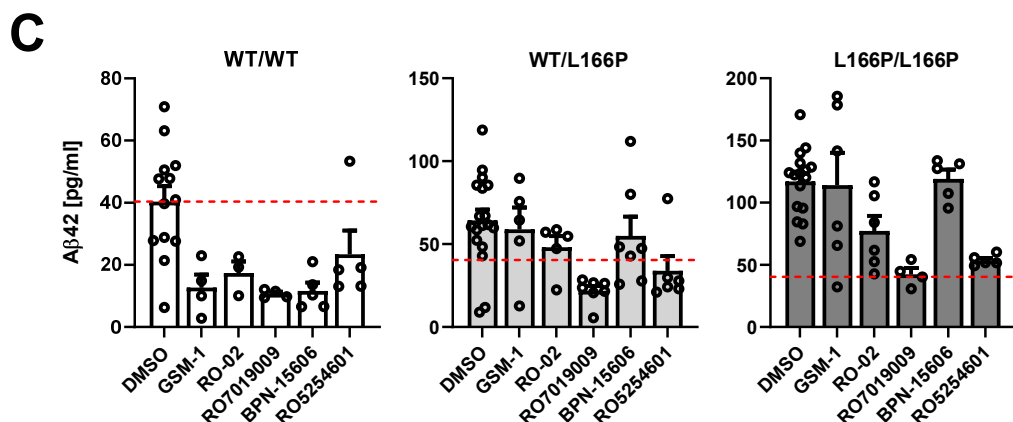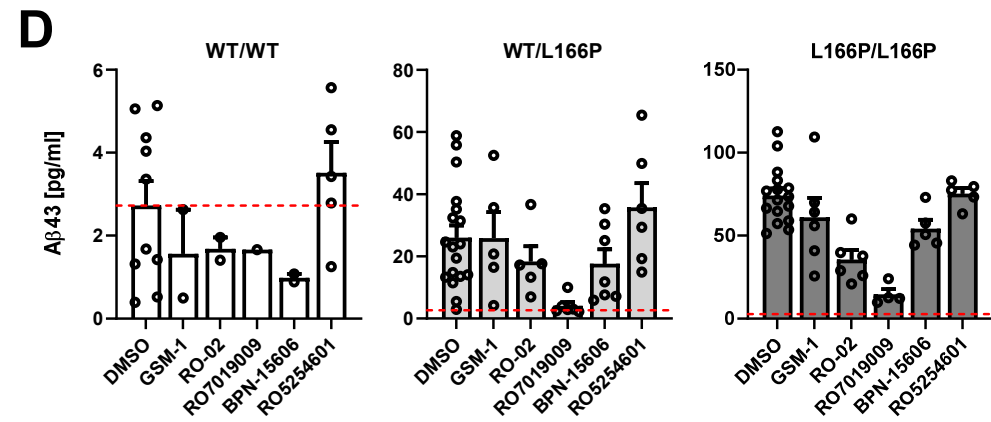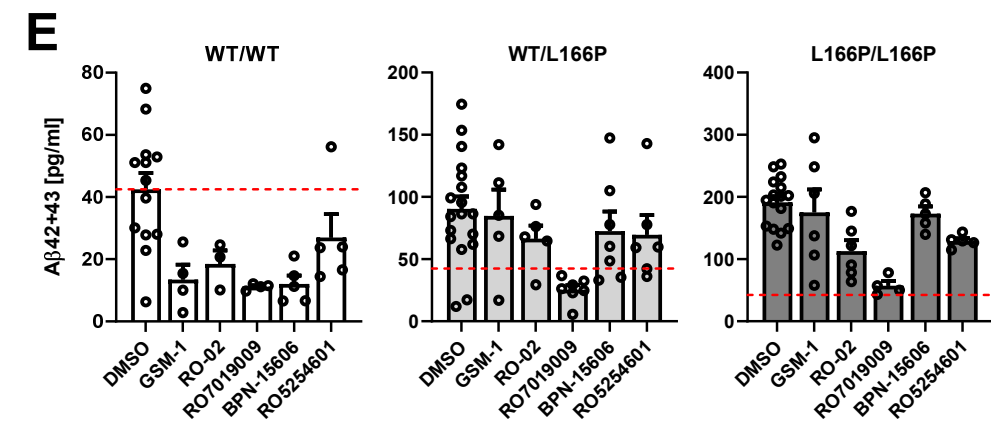

Supplement: Supplementary file 2 — Supplementary Material 2: Figure S2. Concentrations of Aβ species secreted by WT or PS1 L166P KI MEF cells treated with different GSMs. (A-E) Measured amounts (pg/ml) of Aβ38 (A), Aβ40 (B), Aβ42 (C), Aβ43 (D) and Aβ42+43 (E) in the medium of WT (WT/WT) and heterozygous (WT/L166P) or homozygous (L166P/L166P) KI MEF cells that were treated with 2.5 µM GSM-1, 500 nM RO-02, 500 nM RO7019009, 360 nM BPN-15606 or 2.5 µM RO5254601 (n = 4–7). Aβ species were measured by species-specific Aβ ELISA (IBL). Data are shown together with the corresponding DMSO controls and are presented as mean + SEM. Dashed red lines highlight the corresponding ratio of the DMSO-treated WT control. Missing data points are due to Aβ levels below the detection limit of the assay. ND; the Aβ species could not be detected in any sample of this condition. [file 13195_2025_1680_MOESM2_ESM.pdf]

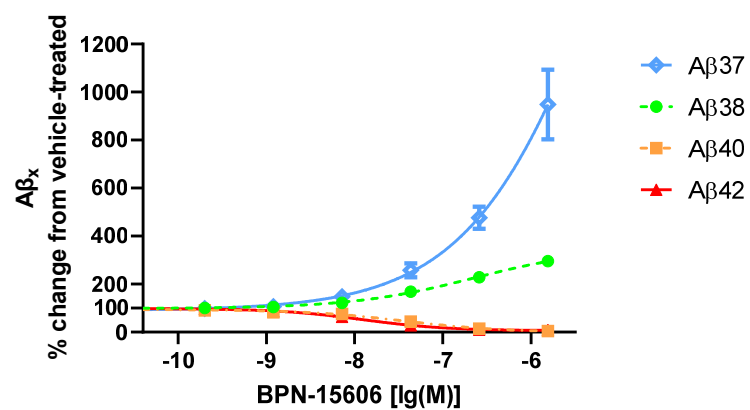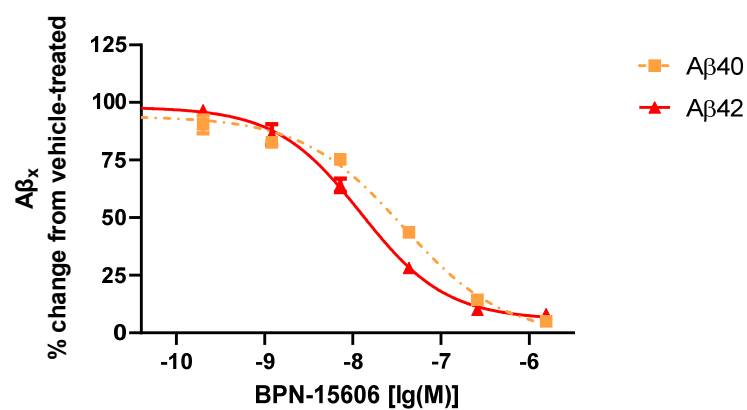

Supplement: Supplementary file 3 — Supplementary Material 3: Figure S3. Dose-response analysis of BPN-15606. Dose-response curves of BPN15606 in HEK293/sw cells measured using the MSD sandwich immunoassay (n = 4–6). In the lower panel, Aβ37 and Aβ38 were excluded to enlarge the effects on Aβ40 and Aβ42. Data are presented as mean ± SEM. [file 13195_2025_1680_MOESM3_ESM.pdf]

**A**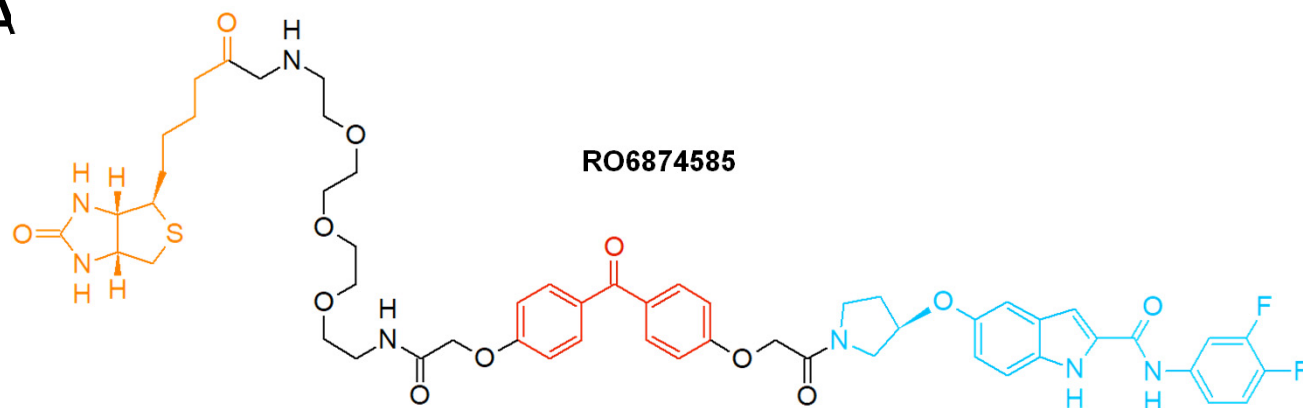**B**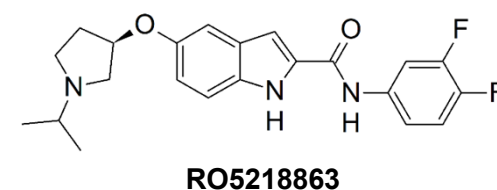**C**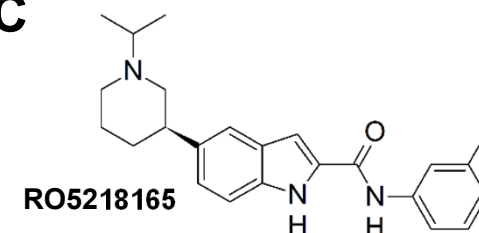**D**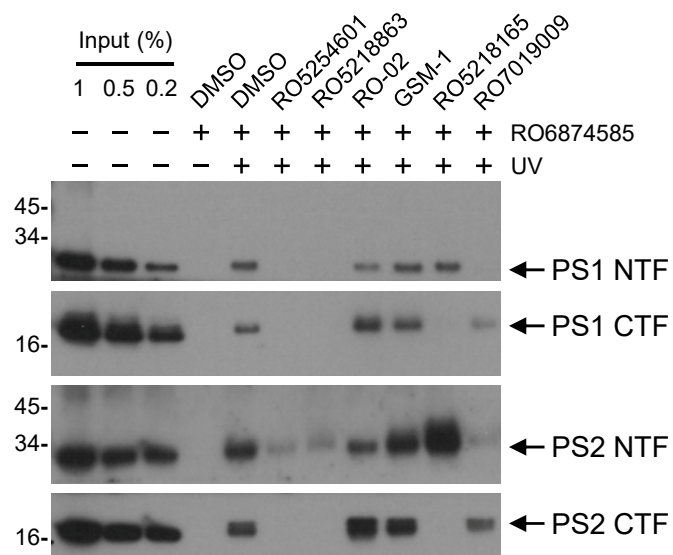**E**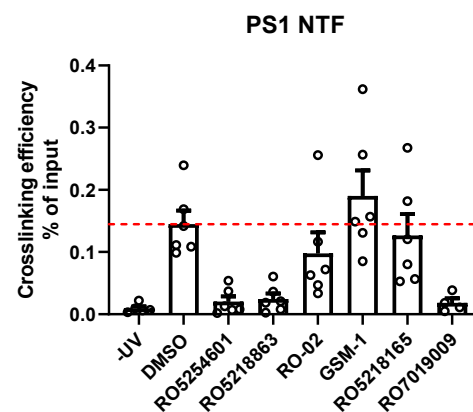**F**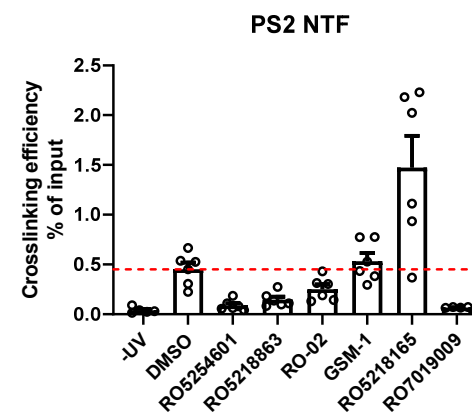**G**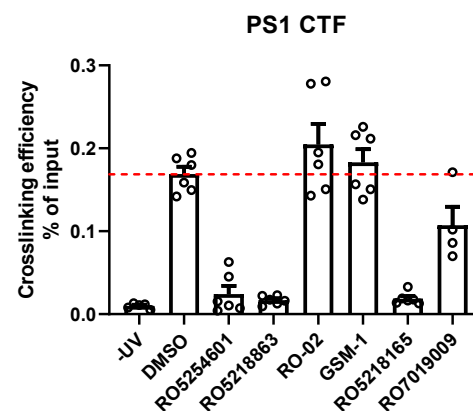**H**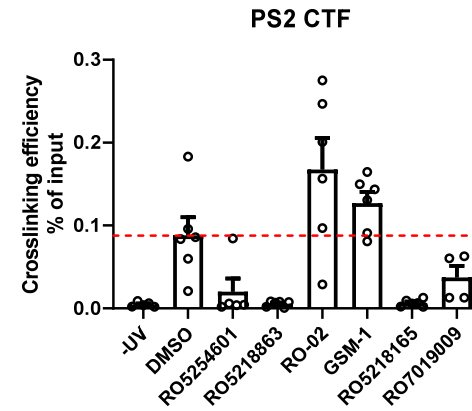**I**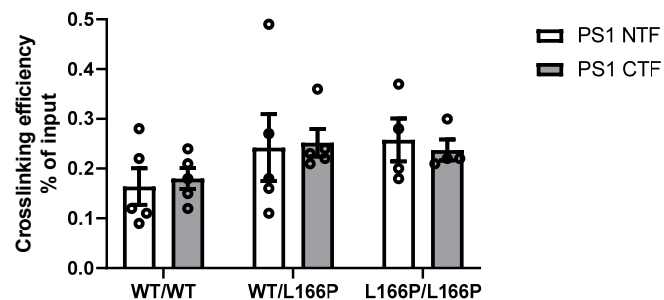

Supplement: Supplementary file 4 — Supplementary Material 4: Figure S4. Indole-type GSMs bind to the presenilin NTF and CTF. (A) Structure of the photocrosslinkable compound RO6874585. Compound moieties are colored in blue (GSM), red (benzophenone) and orange (biotin). (B) Structure of RO5218863, the parental compound of the crosslinkable derivate RO6874585. (C) Structure of the additional indole-type GSM RO5218165. (D) Immunoblot analysis of photoaffinity-labeling experiments with the indole-type GSM RO6874585. Competition of crosslinking (DMSO control) was analysed using a 100x excess of RO5254601, the parental compound RO5218863, RO-02, GSM-1, RO5218165 or RO7019009. Samples that were not UV-irradiated were loaded to control for specificity. (E-H) Quantification of crosslinking efficiencies of PS1 NTF (E), PS2 NTF (F), PS1 CTF (G) and PS2 CTF (H) in HEK293 cell membranes in the presence of the indicated GSM (n = 4–6).(I) Quantification of crosslinking efficiencies of PS1 NTF and PS1 CTF in WT or PS1 L166P KI MEF cell membranes (n = 4–5). Data in (E-I) are presented as mean + SEM. Dashed red lines (E-H) highlight the crosslinking efficiency of the DMSO vehicle-treated control. [file 13195_2025_1680_MOESM4_ESM.pdf]

**A**

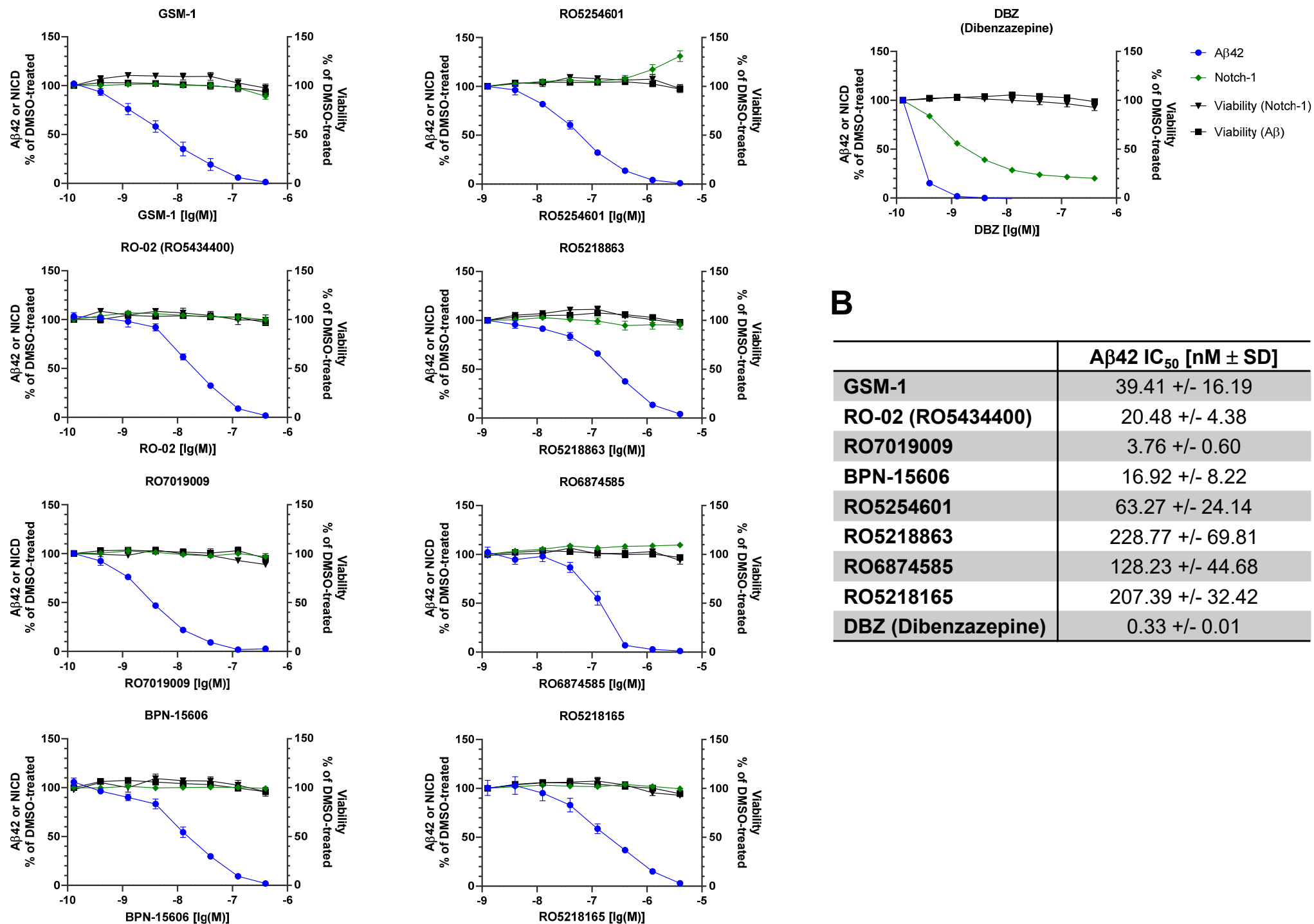

**B**

|                     | Aβ42 IC <sub>50</sub> [nM ± SD] |
|---------------------|---------------------------------|
| GSM-1               | 39.41 +/- 16.19                 |
| RO-02 (RO5434400)   | 20.48 +/- 4.38                  |
| RO7019009           | 3.76 +/- 0.60                   |
| BPN-15606           | 16.92 +/- 8.22                  |
| RO5254601           | 63.27 +/- 24.14                 |
| RO5218863           | 228.77 +/- 69.81                |
| RO6874585           | 128.23 +/- 44.68                |
| RO5218165           | 207.39 +/- 32.42                |
| DBZ (Dibenzazepine) | 0.33 +/- 0.01                   |

Supplement: Supplementary file 5 — Supplementary Material 5: Figure S5. Potencies of the GSMs used. (A) The Aβ42-lowering effects of the GSMs used in this study were additionally characterised in dose-response curves on the H4/sw cell line, measuring Aβ42 levels after over-night incubations with an Aβ42-AlphaLisa. Effects of compounds on Notch1 processing were recorded using a Notch1 reporter assay. Effects of compounds on cell viability were recorded using the same compound concentrations and incubation conditions as in the Aβ42 and Notch1 assays, respectively. The GSI DBZ was included as reference. Means ± SEM of 4–7 independent experiments with 2 technical replicates each are shown. (B) IC50 values for Aβ42 calculated from the experiments in (A). [file 13195_2025_1680_MOESM5_ESM.pdf]

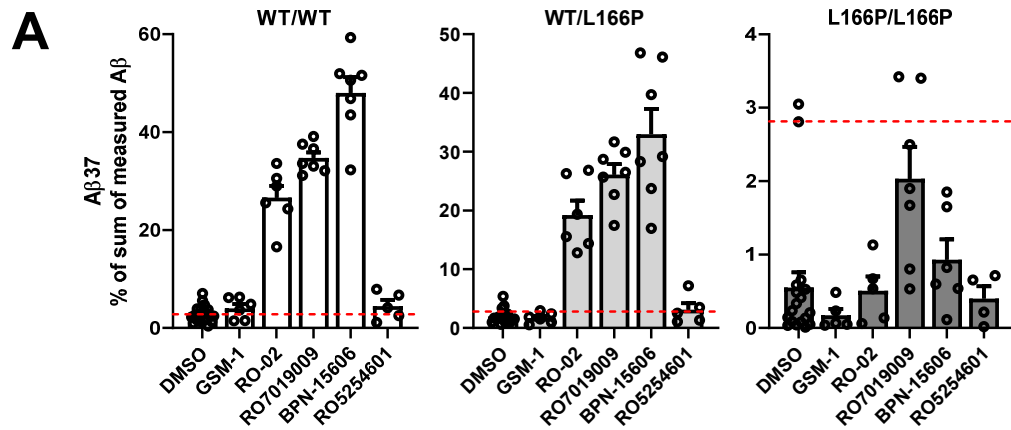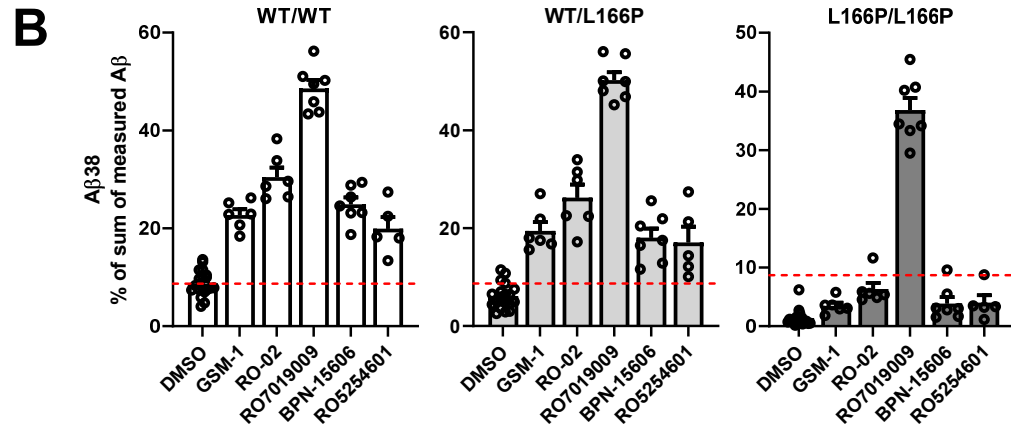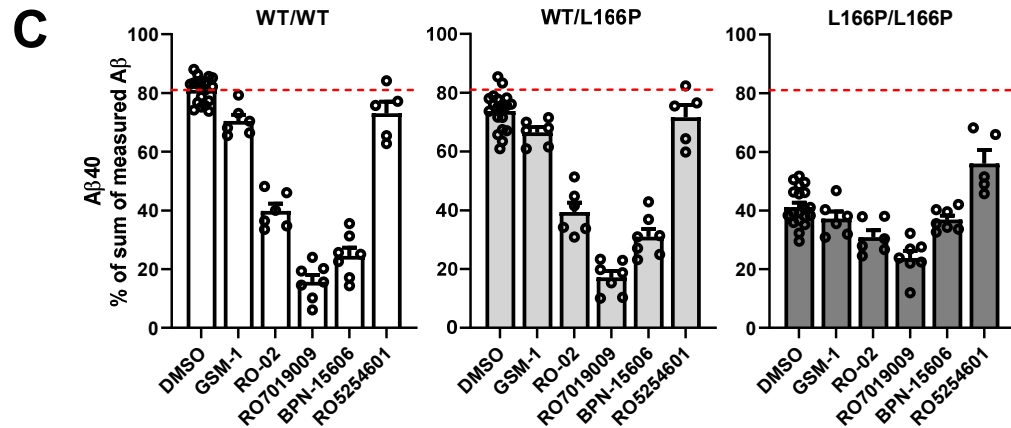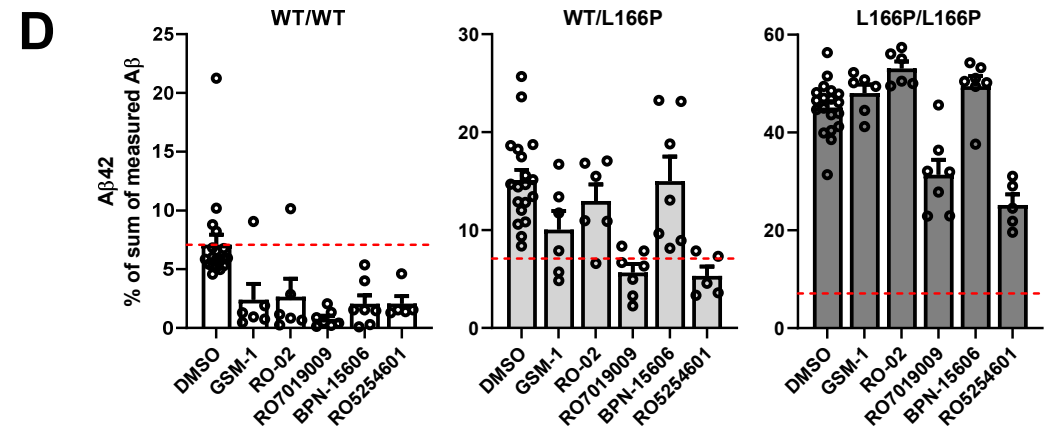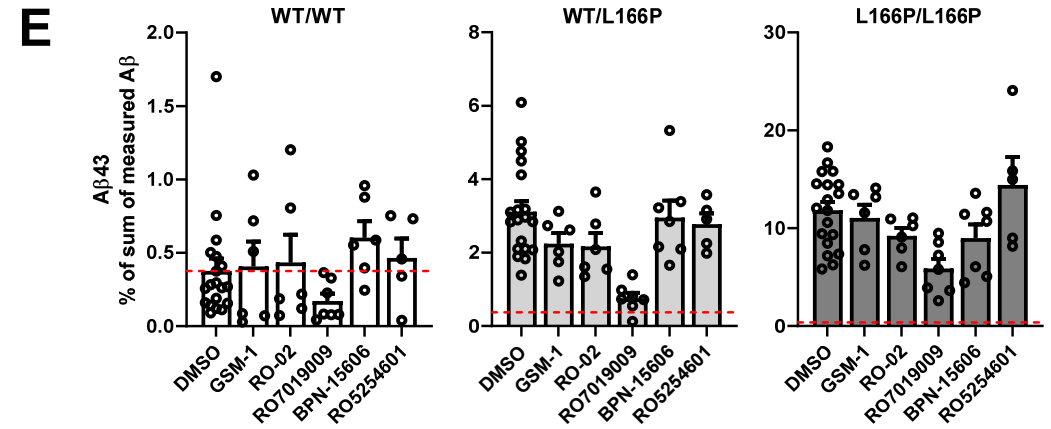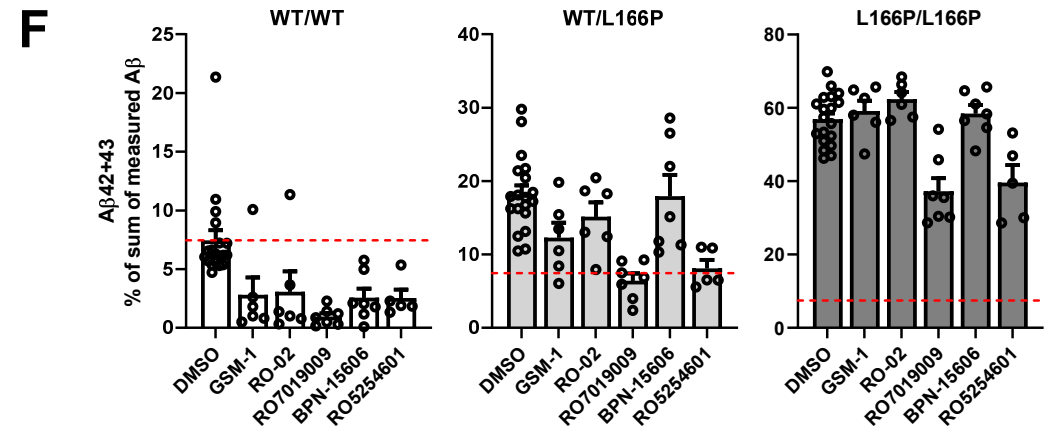

Trambauer et al., Supplementary Material 6: Figure S6.

Supplement: Supplementary file 6 — Supplementary Material 6: Figure S6. Ratios of Aβ species secreted by WT or PS1 L166P KI MEF cells as analysed by Tris-Bicine urea SDS-PAGE. (A-F) Ratios of Aβ37 (A), Aβ38 (B), Aβ40 (C), Aβ42 (D), Aβ43 (E) and combined ratio of Aβ42+43 (F) expressed as % of the sum of measured Aβ (37+38+40+42+43) from WT (WT/WT) and heterozygous (L166P/WT) or homozygous (L166P/L166P) KI MEF cells treated with the depicted GSMs (GSM-1 and RO5254601 at 2.5 µM, RO-02 and RO7019009 at 500 nM and BPN-15606 at 360 nM, respectively) (n = 5–7). Aβ species were separated on Tris-Bicine urea gels and analysed by immunoblotting. The calculated ratios are shown together with those of the corresponding DMSO controls and presented as mean + SEM. Dashed red lines highlights the corresponding ratio of the DMSO vehicle-treated WT control. Missing data points are due to Aβ signals that could not be quantified. [file 13195_2025_1680_MOESM6_ESM.pdf]

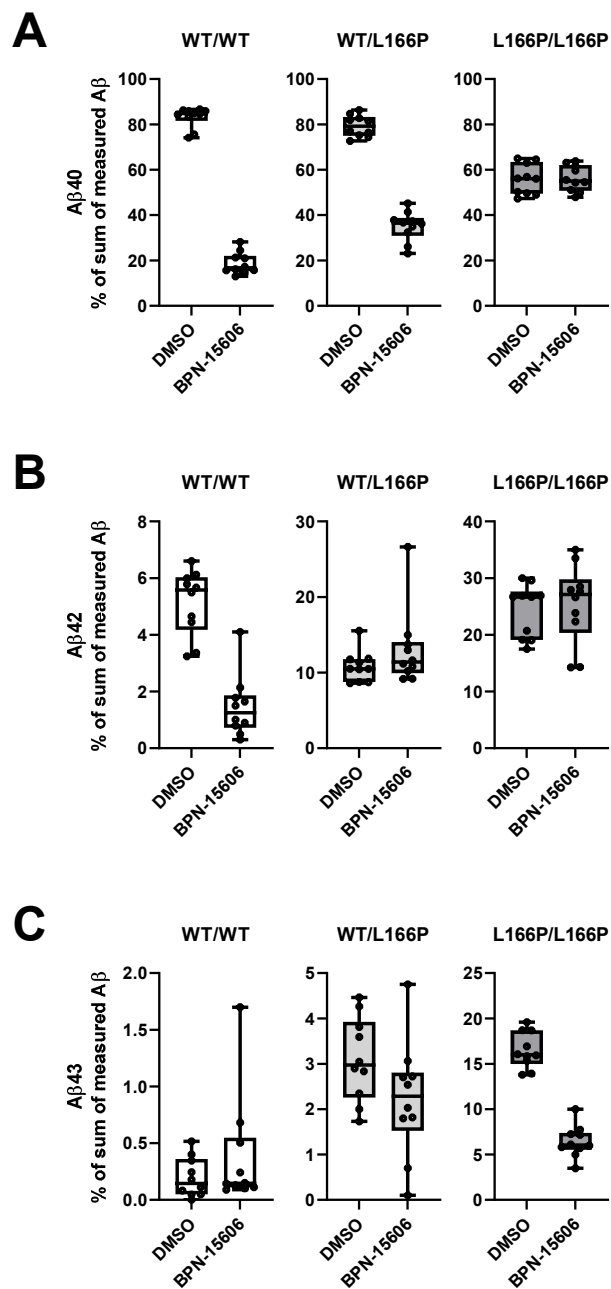

Supplement: Supplementary file 7 — Supplementary Material 7: Figure S7. Effect of an increased dose of BPN-15606 on the secretion of Aβ in WT and PS1 L166P KI MEF cells. (A-C) Ratios of Aβ40 (A), Aβ42 (B) and Aβ43 (C) expressed as % of the sum of measured Aβ (37+38+40+42+43) from WT (WT/WT) and heterozygous (L166P/WT) or homozygous (L166P/L166P) KI MEF cells treated with DMSO or 500 nM BPN-15606 (n = 10). Aβ species were separated on Tris-Bicine urea gels and analysed by immunoblotting. The calculated ratios are presented as box-whiskers-plot. [file 13195_2025_1680_MOESM7_ESM.pdf]

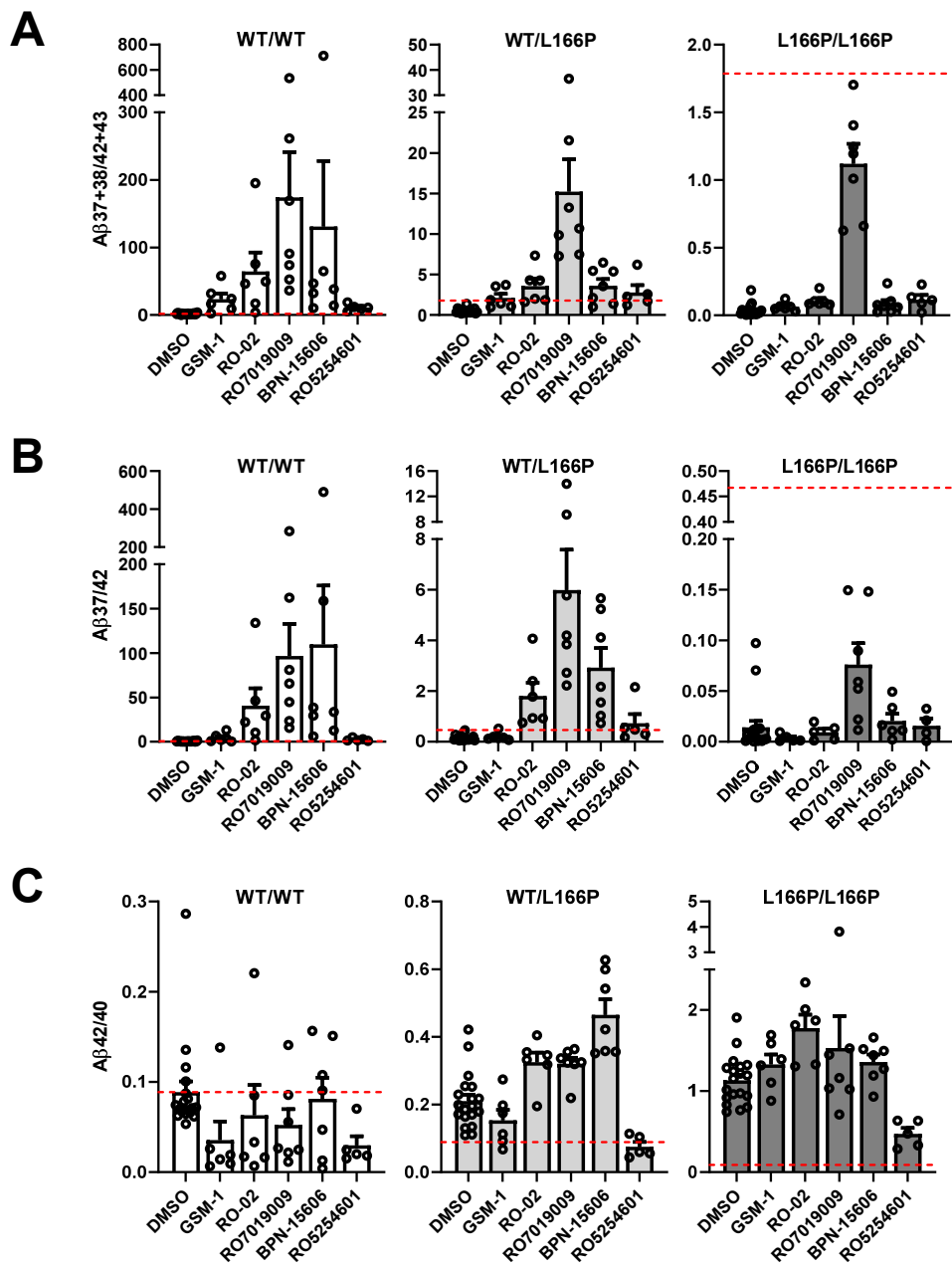

Trambauer et al., Supplementary Material 8: Figure S8.

Supplement: Supplementary file 8 — Supplementary Material 8: Figure S8. Ratios between different Aβ species. (A) Ratio of the short Aβ37/Aβ38 species representing direct or indirect products of the longer pathogenic Aβ42/Aβ43 species (n = 5–7). (B) Ratio of Aβ37 to Aβ42 (n = 5–7). (C) Ratio of Aβ42 to Aβ40 (n = 5–7). Aβ species were separated on Tris-Bicine urea gels and analysed by immunoblotting. The values in (A-C) were calculated from the ratios of the single Aβ species. Data are shown together with the corresponding DMSO controls and presented as mean + SEM. The red dashed line highlights the corresponding ratio of the DMSO vehicle-treated WT control. [file 13195_2025_1680_MOESM8_ESM.pdf]

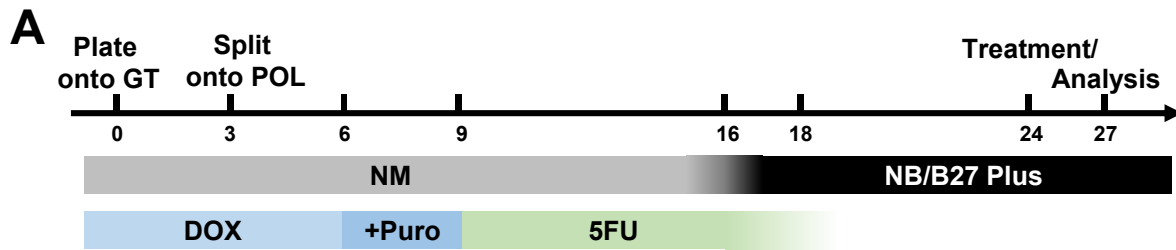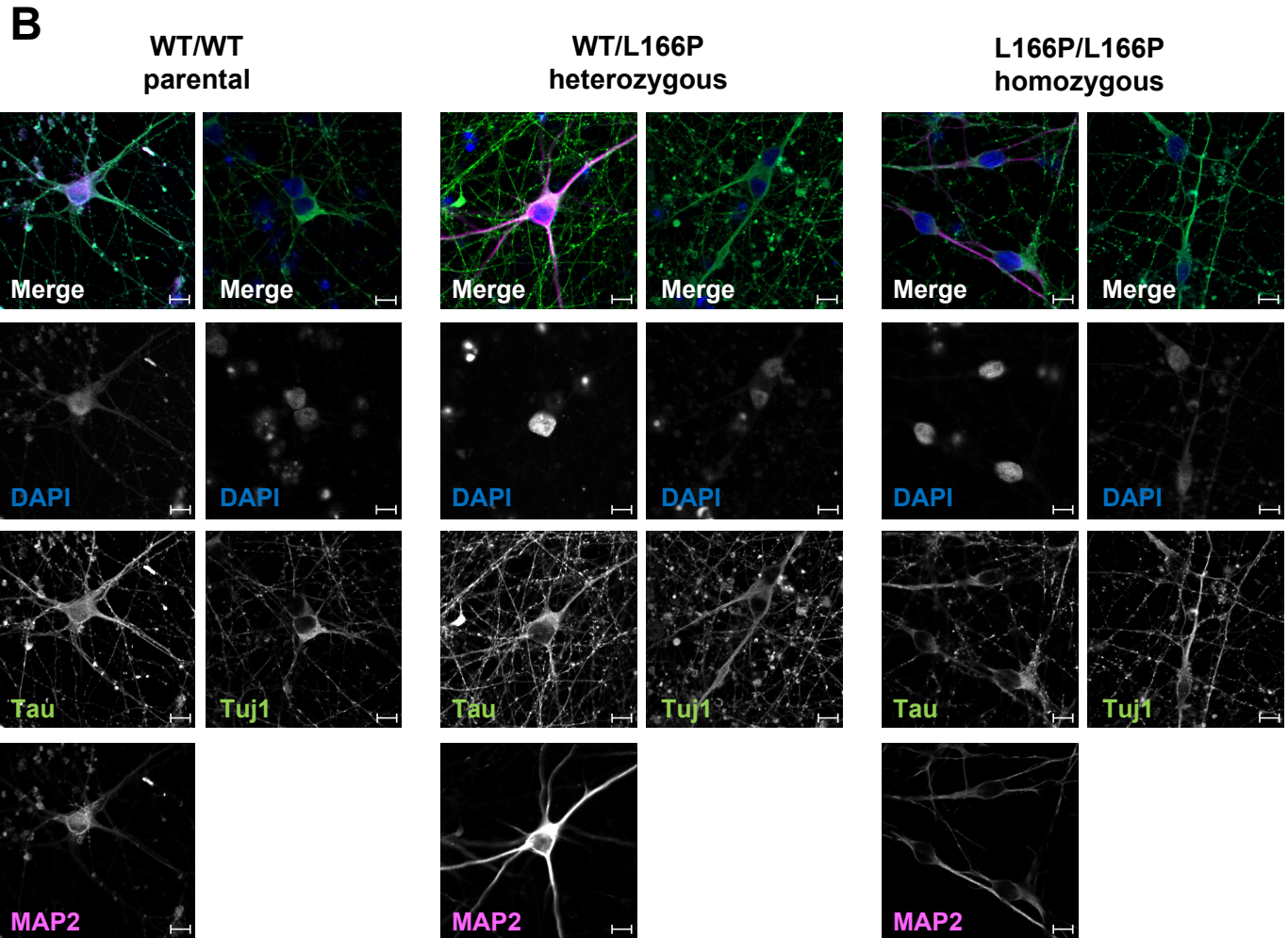

63x

Supplement: Supplementary file 10 — Supplementary Material 10: Figure S10. Ngn2-expressing iPSCs can be differentiated into cortical neurons after doxycycline induction. (A) Schematic workflow of the differentiation protocol (DOX, doxycycline; Puro, puromycine; 5FU, 5-fluorouracil), see Materials and methods for details. (B) Single channel and merged immunofluorescence stainings (DAPI (blue), Tau (green), MAP2 (pink) and Tuj1 (green)) of differentiated neurons at day 27. The merged picture is also shown in Fig. 5B. Magnification: 63x; Scalebar: 10 µm. [file 13195_2025_1680_MOESM10_ESM.pdf]

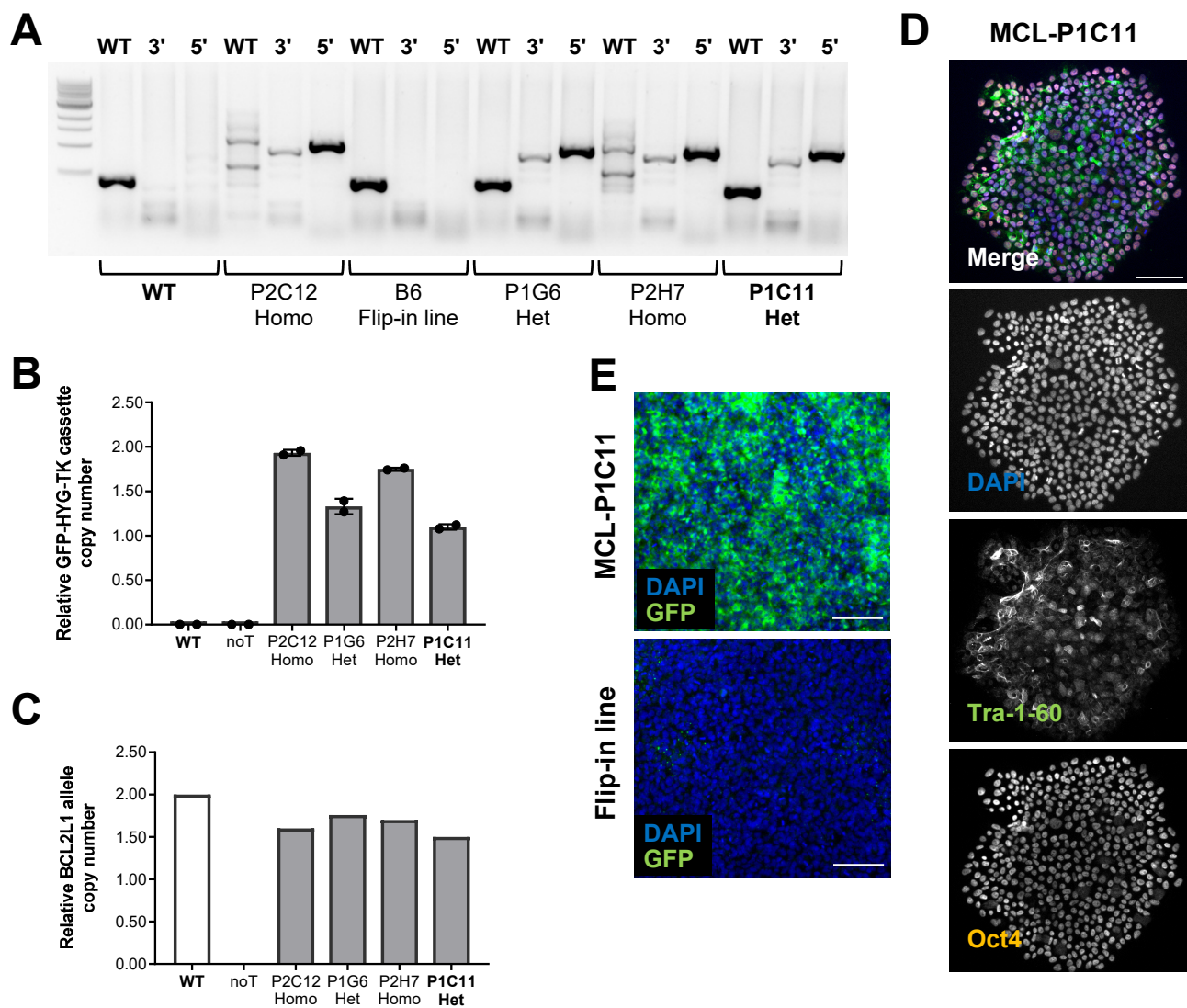

Trambauer et al., Supplementary Material 11: Figure S11.

Supplement: Supplementary file 11 — Supplementary Material 11: Figure S11. Quality control and validation of the flippable master cell line (MCL) iPSCs. (A, B) PCR-genotyping (A) of correct integration of the GFP-HYG-TK cassette in the AAVS1 locus of the MCL candidates in the selected cell clones. The B6 cell line was used to control the primer specificity for the cassette (A). MCLs were characterised as homozygous (Homo) or heterozygous (Hetero) based on the presence of a WT band or the amplification product of the 5′/3′ junction regions of the cassette (A), which was further confirmed by the relative GFP-HYG-TK cassette copy number (B). (C) Clones were screened for genetic alterations in the anti-apoptotic gene BCL2L1. (D) Representative immunohistochemistry image of a MCL clone expressing the pluripotency markers Tra-1-60 (cytoplasmic) and Oct4 (nuclear). Scale bar 100 μm. (E) Representative immunocytochemistry images of GFP expression in undifferentiated MCL cells (top) or after recombinase-mediated cassette exchange by which the GFP-HYG-TK cassette of the MCL is exchanged by the DOX-induced transcription factors resulting in a loss of GFP expression (bottom). Data in (B) are represented as mean ± SD of 2 independent experiments. Data in (C) are represented as the mean of 2 technical replicates. [file 13195_2025_1680_MOESM11_ESM.pdf]

**A**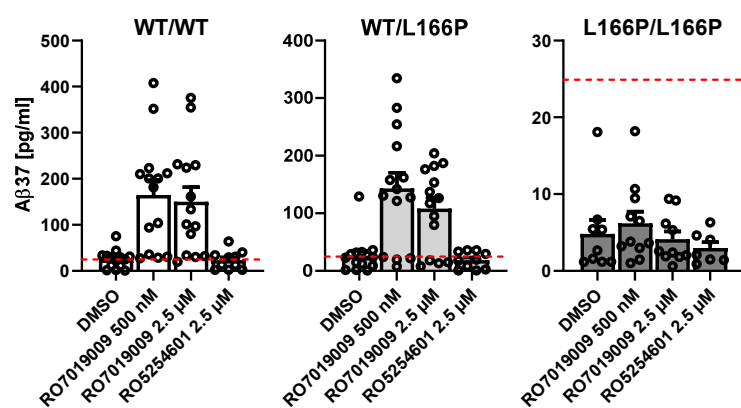**B**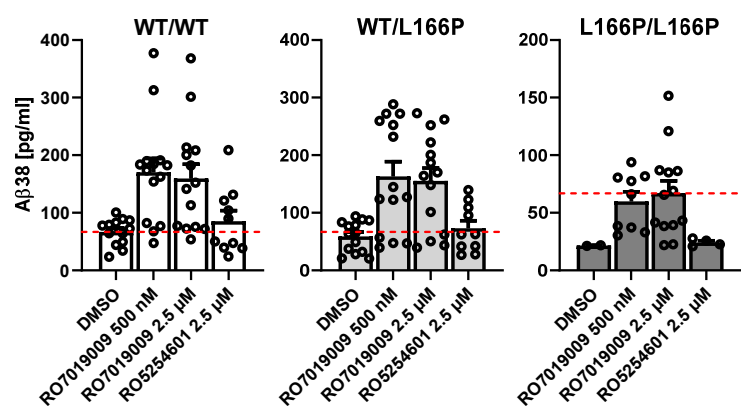**C**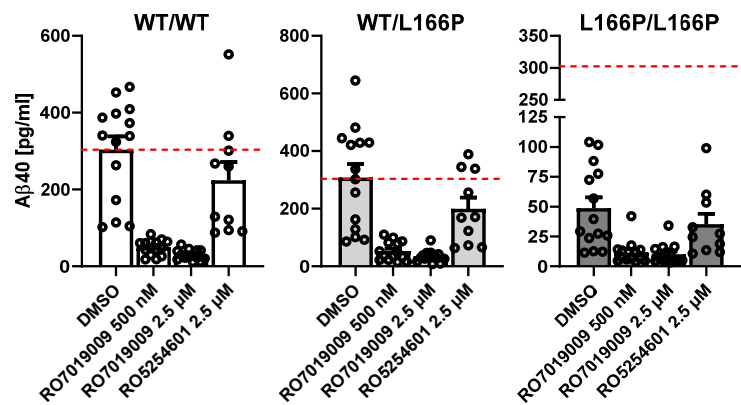

Supplement: Supplementary file 12 — Supplementary Material 12: Figure S12. Levels of Aβ secreted by iPSC-derived neurons. (A-C) Measured amounts (pg/ml) of Aβ37 (A), Aβ38 (B) and Aβ40 (C) in the medium of WT (WT/WT), heterozygous (WT/L166P) or homozygous (L166P/L166P) PS1 L166P KI neurons that were treated with DMSO, RO7019009 (500 nM and 2.5 µM) or RO5254601 (2.5 µM) (n = 10–14). Aβ species were measured by species-specific Aβ ELISA (IBL) (Aβ38 and Aβ40) or electrochemiluminescence immunoassay (MSD, Aβ37) and are presented as mean + SEM. The dashed line highlights the levels of the DMSO vehicle-treated WT control. Missing data points are due to Aβ levels below the detection limit of the assay. [file 13195_2025_1680_MOESM12_ESM.pdf]
