# Supplementary material for: γ-Secretase modulator resistance of an aggressive Alzheimer-causing presenilin mutant can be overcome in the heterozygous patient state by a set of advanced compounds
Source: Alzheimers Res Ther. 2025 Feb 19;17:49. doi: 10.1186/s13195-025-01680-3 (PMC11837686; doi:10.1186/s13195-025-01680-3)
Supplement: Supplementary file 9 — Supplementary Material 9: Figure S9. Quality control and validation of edited Ngn2-inducible iPSCs containing one or two PS1 L166P alleles. (A) List of predicted most likely off-target sites of the used gRNA. None of the sites showed any alteration after editing. (B, C) Analysis of CRISPR-mediated on-target effects by qgPCR quantitation of allele copy number (B) and Sanger sequencing of SNPs near the edited locus in WT and PS1 L166P iPSC lines (C) shows maintenance of both alleles after editing. (D) Single channel and merged immunofluorescence stainings of pluripotency markers Tra-1-60, Oct4, SSEA4, and NANOG in edited iPSCs. Scalebar: 50 µm. [file 13195_2025_1680_MOESM9_ESM.pdf]

A

ID	Sequence	Mismachtch Position	Mismatch Count	MIT Score	CFD Score	Location	Location Description	Indels detected
gRNA	TAGAGATGATATAATAAGCCAGG	Target						
OT1	GGGAGATAATAAAATAAGCCAGG	**.....*...*........	4	0.70	0.61	2:153724855:153724877:1	intergenic:AC009969.1-UBQLN4P2	None
OT2	AAAAAATGATAAAATAAGCCAGG	*.*.*......*........	4	0.69	0.52	21:46695806:46695828:1	intron:POFUT2	None
OT3	AAGAAAGGATATAATAAGCCAGG	*...*.*............	3	1.74	0.51	21:31259021:31259043:-1	intergenic:GRIK1-AS1-GRIK1	None
OT4	GTGAAATGATAAAATAAGCCTGG	**..*......*........	4	0.70	0.48	5:178143770:178143792:1	intron:ZNF354A	None
OT5	TAGAGATAAAAAAAAAAGCCAGG	.......*.*.*..*.....	4	0.17	0.40	2:175393048:175393070:1	intergenic:AC010894.4-AC018890.6	None
OT6	TTAAGATGATAGAATAAGCCCGG	.**........*........	3	1.37	0.27	9:12844099:12844121:1	intergenic:LURAP1L-Y_RNA	None
OT7	GAGACATTAGATAATAAGCCAGG	*...*..*.*.........	4	1.32	0.23	1:175940964:175940986:-1	intergenic:SCARNA3-RFWD2	None
OT8	TATAAATGATAGAATAAGCCAGG	..*.*......*........	3	1.30	0.22	8:24510893:24510915:1	intron:RP11-624C23.1	None
OT9	TGTTGATGAAATAATAAGCCAGG	.***.....*.........	4	1.24	0.22	13:90205830:90205852:-1	intergenic:RP11-75N6.3-LINC00353	None

B

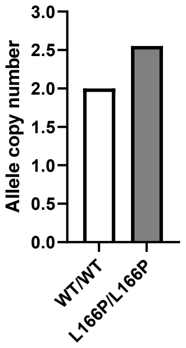

C

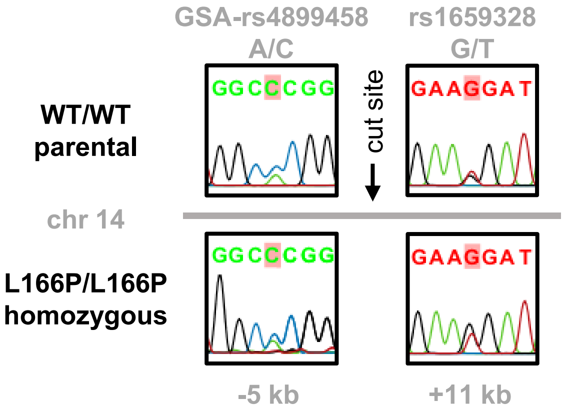

D

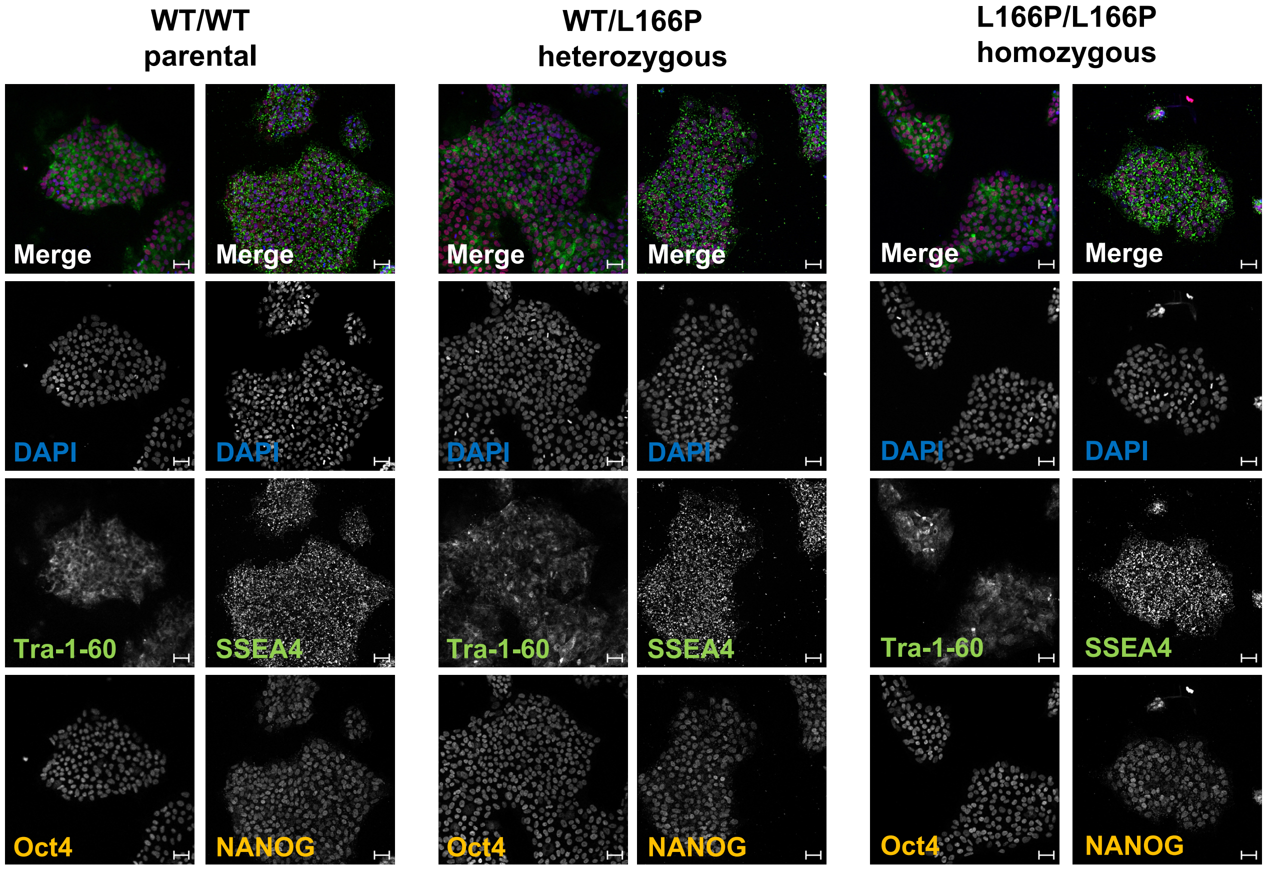

Trambauer et al., Supplementary Material 9: Figure S9.
