# Supplementary material for: γ-Secretase modulator resistance of an aggressive Alzheimer-causing presenilin mutant can be overcome in the heterozygous patient state by a set of advanced compounds
Source: Alzheimers Res Ther. 2025 Feb 19;17:49. doi: 10.1186/s13195-025-01680-3 (PMC11837686; doi:10.1186/s13195-025-01680-3)
Supplement: Supplementary file 13 — Supplementary Material 13: Table S1. Overview of IC50 (Aβ42) values and GSM concentrations tested. [file 13195_2025_1680_MOESM13_ESM.docx]

**Supplementary Material 13: Table S1. Overview of IC_50_ (Aβ42) values and GSM concentrations tested.**

|  | **Lower potency GSMs** | | **Higher potency GSMs** | | |
| --- | --- | --- | --- | --- | --- |
| **Compound** | **GSM-1** | **RO5254601** | **RO-02** | **RO7019009** | **BPN-15606** |
| **IC_50_** | 180 nM ^[25]^ | 380 nM ^a^ | 15 nM ^[49]^ | 14 nM ^[16]^ | 12 nM ^a^ |
| **IC_50_ multiples** | 14x | 6.6x | 33x | 36x / 179x ^b^ | 30x / 42x ^c^ |
| **Concentration** | 2.5 µM | 2.5 µM | 0.5 µM | 0.5 / 2.5 µM ^b^ | 360 / 500 nM ^c^ |
| ^a^ This study.  ^b^ Additional concentration of RO7019009 used in neuronal cells.  ^c^ Additional concentration of BPN-15606 used in MEF cells. | | | | | |
|  | | | | | |
