# Supplementary material for: γ-Secretase modulator resistance of an aggressive Alzheimer-causing presenilin mutant can be overcome in the heterozygous patient state by a set of advanced compounds
Source: Alzheimers Res Ther. 2025 Feb 19;17:49. doi: 10.1186/s13195-025-01680-3 (PMC11837686; doi:10.1186/s13195-025-01680-3)
Supplement: Supplementary file 14 — Supplementary Material 14. Supplementary Information. Indole-type GSMs. [file 13195_2025_1680_MOESM14_ESM.pdf]

Supplementary Material 14: Supplementary Information

Indole-type GSMs – Indole-type GSMs such as 5-(1-Acetyl-piperidin-3-yl)-1*H*-indole-2-carboxylic acid (3,5-difluoro-phenyl)-amide (RO5254601) and 5-((R)-1-Isopropyl-piperidin-3-yl)-1*H*-indole-2-carboxylic acid (3-fluoro-phenyl)-amide (RO5218165) were prepared as described in the Roche patent WO2014/060386. In more detail, preparation of RO5254601 (example 46) is on page 58 in the patent, while preparation of the racemic precursor of RO5218165 (example 52) is mentioned on page 63. Chiral separation of the enantiomer, precursor of RO5218165, was performed using a chiral column and the data for the R enantiomer or RO5218165 (example 112) is on page 97 (Table 8) on the mentioned patent. 5-((R)-1-Isopropyl-pyrrolidin-3-yloxy)-1H-indole-2-carboxylic acid (3,4-difluoro-phenyl)-amide (RO5218863) and its photocrosslinkable derivative N-(3,4-difluorophenyl)-5-((R)-1-(2-(4-(4-(2,16-dioxo-20-((3aS,4S,6aR)-2-oxohexahydro-1H-thieno[3,4-d]imidazol-4-yl)-6,9,12-trioxa-3,15-diazaicosyloxy)benzoyl)phenoxy)acetyl)pyrrolidin-3-yloxy)-1H-indole-2-carboxamide RO6874585 were synthesised as described on the schemes below.

Preparation of 5-((R)-1-Isopropyl-pyrrolidin-3-yloxy)-1H-indole-2-carboxylic acid (3,4-difluoro-phenyl)-amide (RO5218863)

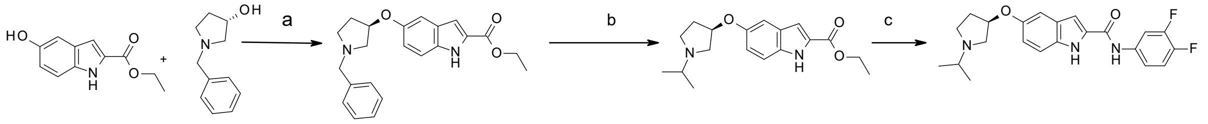

CAS 24985-85-1

(S)-3-Hydroxy-1-benzylpyrrolidine
CAS 101385-90-4

a) Ph_3P (1.5 eq), DIAD (1.5 eq), THF, 56%; b) i) H_2/Pd (10%) (1 eq) in EtOH/AcOH; ii) Iodo- Propane (1.5 eq), K_2CO_3 (2 eq) in DMF, 50°C, 48%; c) Me_3Al (2M in heptane) (4 eq), difluoroaniline (3 eq), dioxane reflux, overnight, 65%.

Preparation of N-(3,4-difluorophenyl)-5-((R)-1-(2-(4-(4-(2,16-dioxo-20-((3aS,4S,6aR)-2-oxohexahydro-1H-thieno[3,4-d]imidazol-4-yl)-6,9,12-trioxa-3,15-diazaicosyloxy)benzoyl)phenoxy)acetyl)pyrrolidin-3-yloxy)-1H-indole-2-carboxamide (RO6874585)

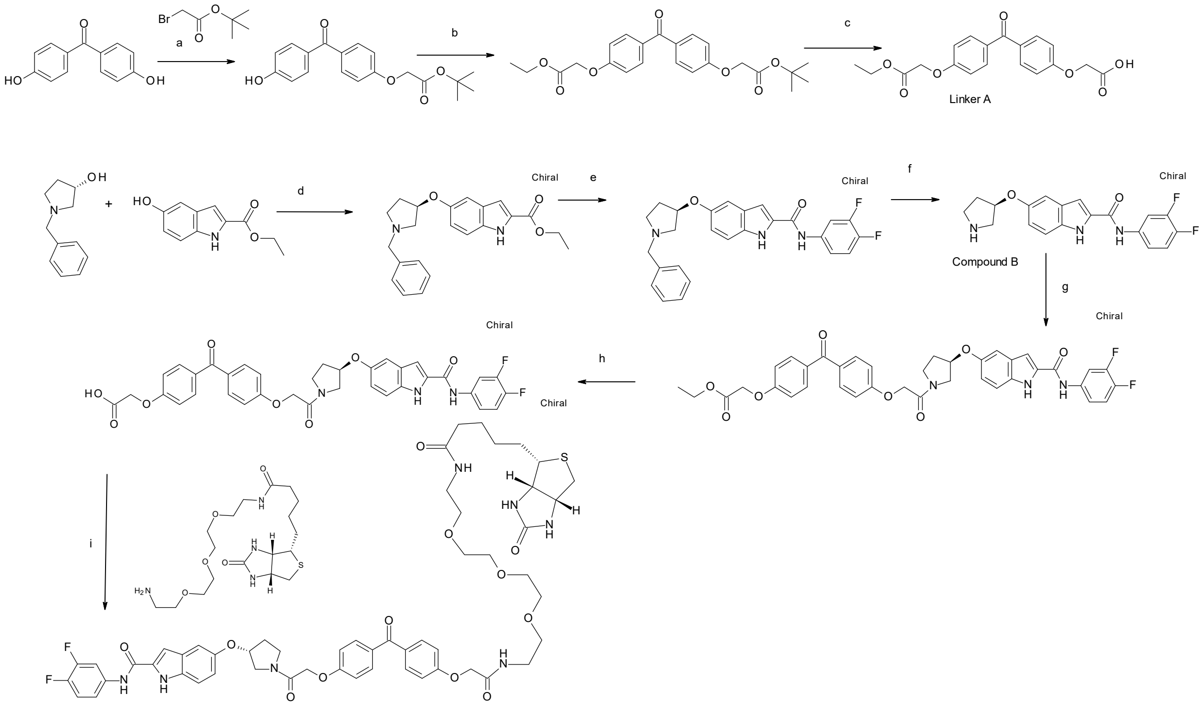

a) Bis (4-hydroxyphenyl)methanone (1 eq), tert-butyl 2-bromoacetate, NaH (1.1 eq), 2h, 0°C, 37%; b) ethyl 2-bromoacetate (1.0 eq), K_2CO_3 (2 eq), CH_3CN/NMP (30/1) (crude 100%); c) formic acid (20 eq), CH_2Cl_2, NMP (30/1), room temperature (r.t) overnight, 82%; d) ethyl 5-hydroxy-1H-indole-2-carboxylate (1 eq), (S)-1-benzylpyrrolidin-3-ol (1.3 eq), Ph_3P (1.5 eq), DIAD (1.5 eq), THF, 56%; e) Me_3Al (4 eq), difluoroaniline (3.2 eq), dioxane reflux, overnight, 69%; f) H_2, Pd/C10% (1 eq), HCl (0.01 eq) in THF, 25% at r.t overnight, 100%; g) 2-(4-(4-(2-ethoxy-2-oxoethoxy)benzoyl)phenoxy)acetic acid (1 eq), indole (1.2 eq), TBTU (1 eq), Hunig´s base (5 eq), overnight, DMF, 53%; h) LiOH (1 eq), THF/H_2O/MeOH (5/2/1), 79%; i) (R)-2-(4-(4-(2-(3-(2-(3,4-difluorophenylcarbamoyl)-1H-indol-5-yloxy)pyrrolidin-1-yl)-2-oxoethoxy) benzoyl)phenoxy) acetic acid (1 eq), N-(2-(2-(2-(2-aminoethoxy)ethoxy)ethoxy)ethyl)-5-((3aS,4S,6aR)-2-oxohexahydro-1H-thieno[3,4-d]imidazol-4-yl)pentanamide (CAS359860-27-8) (1 eq)), N-Methylmorpholine (3 eq), HBTU (1.5 eq), DMF, overnight, 80%.
